# Integrative Single-Cell and Spatial Transcriptomics Analysis Reveals ECM-remodeling Cancer-associated Fibroblast-Derived POSTN as a Key Mediator in Pancreatic Ductal Adenocarcinoma Progression

**DOI:** 10.7150/ijbs.108618

**Published:** 2025-05-27

**Authors:** Yifan Wu, Shuquan Li, Hao Yu, Sha Zhang, Liang Yan, Xiaoya Guan, Wei Xu, Zhen Wang, Ang Lv, Xiuyun Tian, Chunyi Hao, Jianhui Wu

**Affiliations:** 1 Key Laboratory of Carcinogenesis and Translational Research (Ministry of Education), Department of Hepato-Pancreato-Biliary Surgery, Peking University Cancer Hospital & Institute, Beijing, China; 2 Department of Critical Care Unit, Shandong Provincial Hospital Affiliated to Shandong First Medical University, Jinan, Shandong, China.

**Keywords:** Pancreatic ductal adenocarcinoma, Cancer-associated fibroblasts, Single-cell RNA sequencing, Heterogeneity, Stroma-tumor crosstalk, POSTN

## Abstract

Pancreatic ductal adenocarcinoma (PDAC) presents significant clinical challenges owing to its dense stroma and complex tumor microenvironment (TME). In this study, large-scale single-cell transcriptomics and spatial transcriptomics (ST) were integrated to dissect the heterogeneity of fibroblasts and their crosstalk with epithelial cells, with a focus on key ligand-receptor interactions. Eight distinct fibroblast subpopulations were identified, among which extracellular matrix (ECM)-remodeling fibroblasts were particularly enriched in tumor tissues and associated with poor prognosis. ECM-remodeling fibroblasts were located at the terminal stage of the fibroblast pseudotime trajectory, and SOX11 was identified as a key transcription factor in this subpopulation. Further analyses revealed that ECM-remodeling fibroblasts can interact with epithelial cells through the POSTN-ITGAV/ITGB5 ligand-receptor axis, a critical pathway that promotes tumor progression. Clinical analyses demonstrated a strong correlation between POSTN expression and poor prognosis in patients with PDAC. Mechanistically, POSTN interacts with integrin ITGAV/ITGB5 on tumor cells, activating the PI3K/AKT/β-catenin pathway and promoting epithelial-mesenchymal transition (EMT) phenotype. Pharmacological inhibition of the POSTN-integrin axis partially reversed these malignant traits, highlighting its potential as a therapeutic target. This study provides new insights into fibroblast heterogeneity and its role in PDAC progression, emphasizing the POSTN-ITGAV/ITGB5 axis as a promising target for therapeutic interventions.

## Introduction

Pancreatic ductal adenocarcinoma (PDAC) is one of the most intractable cancers worldwide 1. Despite advancements in diagnostics and treatments over recent decades, the 5-year survival rate remains below 9%.^1^ Surgical resection, the primary curative option, is feasible in only 10-15% of patients, and most suffer from metastasis owing to the lack of effective therapies 2. This poor prognosis is largely attributed to the extensive stromal components and tumor heterogeneity in PDAC. The complex and intricate interactions between stromal components and heterogeneous cancer cells significantly hinder our understanding of tumor progression and metastasis. Improving clinical outcomes demands a detailed understanding of stromal heterogeneity and its crosstalk with epithelial cells.

Cancer-associated fibroblasts (CAFs) constitute the predominant component of the PDAC stroma and play a pivotal role in tumor progression. The heterogeneity of CAFs is manifested across diverse levels, from cellular level to microenvironmental and regional level, all of which contribute to their functional diversity 3. Recent single-cell transcriptomic technologies have enabled the characterization of CAFs across various tumors 4-6, including PDAC 7-9. While most studies have focused on descriptive profiling, detailed investigations into CAF-tumor cell crosstalk and clinically relevant targets remain to be further explored. Furthermore, the small sample sizes in these studies have impeded comprehensive understanding of CAFs and cancer cell subpopulations, particularly rare subtypes. Therefore, large-scale integrated single-cell transcriptomic datasets are urgently required to unravel CAF heterogeneity and complex interactions.

CAFs primarily interact with epithelial cells via ligand-receptor interactions, either through soluble ligands binding to receptors or direct cell-cell adhesions 10. Such ligand-receptor communication is deeply involved in tumorigenesis, tumor progression, and therapy resistance 11. For instance, in PDAC, CAF-derived thrombospondin 2 (THBS2) drives tumor progression via integrin αvβ3/CD36-mediated signaling 12. Similarly, in esophageal squamous cell carcinoma (ESCC), CAF-derived collagen I induces radioresistance via the collagen I-integrin axis 13. Understanding the key ligand-receptor interactions in CAF-tumor crosstalk holds significant potential for advancing therapeutic development. However, mapping these signaling pathways at a single-cell level and validating their clinical significance with patient samples and functional assays still remains to be further explored in PDAC research.

This study integrated 66 single-cell transcriptome samples as a discovery cohort supplemented with a partial external validation cohort and spatial transcriptomics (ST) for spatial context. This approach allowed us to delineate the heterogeneity of CAFs and epithelial cells in PDAC. We identified the POSTN-ITGAV/ITGB5 axis as a key mediator in PDAC progression. Clinical analyses revealed a correlation between CAF-derived POSTN and poor outcomes. Mechanistically, functional validation revealed that ECM-remodeling CAF-derived POSTN promotes differentiation of PDAC cells into the epithelial-mesenchymal transition (EMT) phenotype through activating the PI3K/AKT/β-catenin signaling pathway via integrin αvβ5. These findings pave the way for novel therapeutic strategies targeting this signaling axis in PDAC.

## Methods and Materials

### scRNA data collection and basic analysis

A discovery cohort of 66 single-cell RNA sequencing (scRNA-seq) samples was assembled from three publicly available datasets (GSE205013^9^, GSE197177^14^, and CRA001160^7^), with clinical information obtained from the corresponding studies (Table S1). Data were analyzed using the Seurat (v5.0.2) R package. Cells were filtered based on the following criteria: (1) 300-8,000 detected genes, (2) UMI count < 40,000, (3) mitochondrial gene percentage < 10%, and (4) hemoglobin gene percentage < 1%. Samples with fewer than 500 cells were excluded from analysis. The retained 66 samples were merged, normalized, and scaled. The top 2000 HVGs were selected for principal component analysis (PCA), with 30 principal components selected for subsequent clustering. Batch effects were corrected using Harmony (v1.2.1). Clustering was performed using the FindNeighbors and FindClusters functions (resolution = 0.5), and clusters were visualized using uniform manifold approximation and projection (UMAP). Cell annotation was conducted manually, based on marker genes from previous studies. Fibroblast subclusters were re-analyzed following the same pipeline. Additionally, a validation cohort of 44 scRNA-seq samples (GSE155698^15^ and CRA001160) was incorporated and analyzed using the same workflow (Table S2).

### Spatial transcriptomic (ST) data collection and cell-type estimation

ST data for PDAC samples were obtained from GSE235315^16^ (Table S3). Individual ST samples were pre-processed using the Seurat. To evaluate the spatial distribution of clusters identified in the scRNA-seq discovery cohort, ST and scRNA-seq expression matrices were integrated using CellTrek (v0.0.94) 17, incorporating spatial coordinate.

### Functional enrichment analysis of single-cell subclusters

Gene Ontology (GO) enrichment analysis for marker genes in each subcluster was performed using clusterProfiler (v4.6.0), with pathways considered significantly enriched at adjusted *P* < 0.05. Gene set variation analysis (GSVA) on hallmark pathways from MSigDB (v2023.2.Hs) was performed using the GSVA package (v1.34.0) to calculate GSVA scores.

### Trajectory analysis

Fibroblast differentiation trajectories were inferred using Monocle2 (v1.3.4) 18 with default parameters. Trends were further validated using Monocle3. Epithelial cell trajectories were constructed using Monocle3 to outline evolutionary trends.

### Regulatory network inference of single-cell subclusters

Regulatory networks and regulon activities were analyzed using pySCENIC (v0.12.1). Transcription factor (TF) activity was assessed by calculating the AUC of genes regulated by each TF, and the regulon-specific score (RSS) for each TF was also computed.

### Identification of epithelial expression programs

We applied non-negative matrix factorization (NMF) to epithelial cells from each sample to identify programs that captured cellular heterogeneity. These methods were similar to those used in previous studies 19. Fourteen high-quality meta-programs (MPs) were identified, with shared cluster genes, completing a list of 50 genes. The MPs were named based on GO enrichment results.

### Subclustering and annotation of epithelial cell subclusters

Epithelial cells were subclustered using the Seurat pipeline, with NMF replacing PCA for dimensionality reduction to identify subclusters based on gene expression modules. Each cell was scored using the above MP geneset via the “AddModuleScore” function. These scores, combined with the sample origin, facilitated the annotation of epithelial cell states and detailed subcluster assessments.

### Copy number variation (CNV) estimation in epithelial cells

CNVs in epithelial cells were inferred using inferCNV (v1.1.3) with default parameters, using control pancreatic epithelial cells as references. CNVs in epithelial cells were analyzed, and the cells were classified as malignant or non-malignant using the mean squared deviation and 90th percentile of CNV scores in normal cells as the threshold 7, 20.

### Analysis of the prognostic relevance of single-cell subclusters

Bulk RNA-seq data and curated clinical data from the TCGA-PAAD cohort were retrieved from the UCSC Xena database website (https://xenabrowser.net/datapages/). To evaluate the prognostic relevance of fibroblast subclusters, the relative abundance of fibroblast subtypes in TCGA-PAAD bulk RNA-seq samples was estimated using the CIBERSORTx algorithm (https://cibersortx.stanford.edu/), which utilizes gene expression deconvolution to infer cell type proportions in bulk transcriptomic data. Patients were stratified into high- and low-infiltration groups based on the median infiltration level of each fibroblast subtype. Kaplan-Meier survival analysis was performed using the survminer (v0.4.9) and survival (v3.3-1) R packages to compare overall survival (OS) between the groups, and statistical significance was assessed using the log-rank test. For epithelial cell subclusters identified in our study, GSVA (v1.46.0) was used to calculate combined expression values for the subtype-specific signatures (the top 30 DEGs). Patients were classified into high- or low-expression groups using the median as the cutoff value. Survival analysis was performed identically as above.

### Cell-cell communications

CellChat (v2.1.2) was used to investigate the interactions between fibroblasts and epithelial subpopulations in malignant and non-malignant samples, with a particular focus on ligand-receptor pairs in which ECM-remodeling fibroblasts as the signaling sources.

### Clinical samples

A total of 173 patients pathologically diagnosed with PDAC were enrolled at the Peking University Cancer Hospital, referred to as the PUCH-PDAC cohort (Table S4), and used for immunohistochemical (IHC) staining. Tissue samples were preserved using formalin-fixed, paraffin-embedded (FFPE) method. All patients provided informed consent, and the study was approved by the Ethics Committee of Peking University Cancer Hospital (protocol no. 2021KT26).

### IHC staining and evaluation

FFPE tissue sections were deparaffinized, rehydrated, and treated with methanol containing 3% hydrogen peroxide to block endogenous peroxidases. Antigen retrieval was performed according to antibody-specific protocols. Sections were incubated with anti-POSTN antibody (Abcam, ab219056, 1:500) and anti-Ki67 antibody (Abcam, ab15580, 1:100) overnight, followed by secondary antibody staining (ZSGB-BIO, PV-6000). DAB was used for chromogenic detection, and the nuclei were counterstained with hematoxylin. Staining was evaluated independently by two pathologists in a double-blind manner, as previously described 12. POSTN staining scores were derived by multiplying the area score (0: ≤ 5%, 1: 5-25%, 2: 26-50%, 3: 51-75%, 4: ≥ 75%) by the intensity score (0: negative, 1+: weak/yellow, 2+: moderate/light brown, 3+: strong/dark brown), yielding final scores ranging from 0 to 12^12^. Expression was classified as negative (0-2), low (3-6), or high (≥ 6) based on the staining index.

### Western blotting

Total protein was extracted from cells, and the concentration was determined using a BCA kit (CWBIO, China). Proteins were separated by SDS-PAGE (EpiZyme, LK308) and transferred onto PVDF membranes. Membranes were blocked in 5% skim milk in Tris-buffered saline-Tween solution. Next, the PVDF membranes were incubated with primary antibodies (Table S5) overnight and secondary antibodies for 1 hour. Finally, a chemiluminescence detection system was used to detect protein bands.

### Isolation of primary CAFs derived from PDAC

Fresh surgical PDAC tissue was collected from the Peking University Cancer Hospital and cut into 2 mm³ pieces for culturing in DMEM/F12 medium supplemented with 15% fetal bovine serum (FBS) for 2-3 weeks. Fibroblasts were isolated based on their high sensitivity to trypsin. CAFs were verified by western blotting for FAP, α-SMA, and E-cadherin expression.

### Cell culture

Human pancreatic cancer cell lines BxPC-3 and PANC-1 were obtained from ATCC (Manassas, VA, USA), whereas human embryonic pancreatic tissue-derived fibroblast CCC-HPE-2 cells were sourced from the National Infrastructure of Cell Line Resources (Beijing, China). BxPC-3 cells were cultured in RPMI 1640 medium supplemented with 10% FBS and 100 U/ml penicillin-streptomycin. PANC-1 and CCC-HPE-2 cells were maintained in Dulbecco's modified DMEM medium (Gibco) supplemented with 10% FBS and 100 U/ml penicillin-streptomycin. All cells were cultured at 37 °C in a humidified incubator with 5% CO₂.

### Lentiviral particle transductions

CCC-HPE-2 cells were transduced with POSTN-overexpressing (oePOSTN) or negative control (NC) lentivirus using the GV703 vector (CMV enhancer-MCS-3FLAG-EF1a-ZsGreen1-T2A-puromycin; GeneChem, China). Transduction was performed according to the manufacturer's instructions. Transduced cells were selected with 2 μg/mL puromycin for 2 weeks, and efficiency was confirmed via western blotting. Primary CAFs were transfected with three POSTN-targeting shRNA lentiviruses or an NC lentivirus using the GV152 vector (hU6-MCS-CMV-Neomycin) to knock down POSTN expression. For primary CAFs, cells were selected with 400 μg/mL G418 for 2 weeks following transduction, while all other procedures were the same as described above.

### Conditioned medium production

An estimated 1 × 10^7^ CAF-oePOSTN, CAF-shPOSTN, and their respective control cells (CAF-NC, pCAF-NC) were cultured to 80% confluence in a serum-free medium for 48 h. The conditioned medium (CM) was collected, centrifuged (4 °C, 1,000 rpm, 5 min), filtered (0.22 μm), and concentrated using a 30 kDa cutoff filter (Millipore, USA) at 4 °C and 2,160 × *g* for 20 min. CM was used immediately or stored at 4 °C for up to 3 days and diluted as needed prior to experiments.

### Cell proliferation and colony formation assays

BxPC-3 cells were pretreated for 24 h with CAF-oePOSTN CM, CAF-NC CM, recombinant human Periostin (rhPOSTN, Novoprotein, CJ39), or integrin αvβ5 receptor inhibitor (MCE, HY-16141). Cells (2,000 cells/well) were seeded in 96-well plates and cultured for 5 d, with 10 μL CCK-8 (Dojindo) added at 24-hour intervals. After 2-3 h of incubation, the absorbance was recorded at 450 nm. For the colony formation assay, 600 BxPC-3 cells were seeded in 6-well plates and cultured for 10 d, with the medium replaced every 4 d. Colonies were fixed with methanol, stained with 0.5% crystal violet, and then quantified using ImageJ software (v1.54f) to calculate the colony formation rate as follows: (number of colonies formed/number of seeding cells) × 100%. PANC-1 cells were pretreated with pCAF-shPOSTN CM or pCAF-NC CM, recombinant human Periostin (rhPOSTN), or integrin αvβ5 receptor inhibitor (MCE, HY-16141). The subsequent experimental treatments and procedures were identical to those for BxPC-3 cells, except that 1,000 PANC-1 cells were seeded per well in 6-well plates for the colony formation assay.

### Wound-healing assays

Cells (1 × 10⁶/well) were seeded in 6-well plates. When confluence was reached, a scratch was made using a 1,000 μL pipette tips. Cells were treated with medium containing 40% CAF CM (for each group) or 500 ng/mL rhPOSTN. Images of wound areas were captured at 0 and 24 h (500× magnification, DMi8; Leica) and analyzed with ImageJ (v1.54f) to calculate the percentage of wound healing as follows: (1 - [current wound area/initial wound area]) × 100%.

### Transwell migration and invasion assays

BxPC-3 cells were pretreated for 24 h with 40% CAF-oePOSTN CM, CAF-NC CM, 500 ng/mL rhPOSTN, or integrin αvβ5 inhibitor (HY-16141), then seeded (4.5 × 10⁴ cells/well) into the upper chambers (Cat. No. 3422, Corning, USA) with 2% FBS. Medium containing 10% FBS was added in the lower chamber as a chemoattractant. After 24 h at 37 °C, the membranes were fixed with 4% paraformaldehyde, stained with 0.1% crystal violet, and cells migrating through the membrane were counted in three random fields per well under a microscope. For the invasion assays, Matrigel (Cat. No. 356237, Corning, USA) was applied to the upper chambers overnight at 37 °C. PANC-1 cells underwent identical treatments with 40% pCAF-shPOSTN CM or pCAF-NC CM, seeded at a density of 2 × 10⁴ cells/well following the same experimental protocol as above.

### Xenotransplant murine model

Six-week-old female BALB/c-Nu mice were purchased from Beijing Vital River Laboratory Animal Technology and randomly divided into two groups (*n* = 5 per group). BxPC-3 cells (2 × 10⁶) mixed with CAF-NC (2 × 10⁶) or CAF-POSTN (2 × 10⁶) were resuspended in 100 μL PBS and subcutaneously implanted into the right armpit of nude mice. Body weights and tumor volumes (calculated as 0.5 × length × width²) were measured every 6 days over a 48-day period. Mice were euthanized, and the tumors were excised, weighed, and analyzed histologically using hematoxylin and eosin (H&E) or IHC staining. All animal procedures complied with the guidelines of the Institutional Animal Care and Use Committee of the Beijing Cancer Hospital (Ethics Approval Number: EAEC 2024-01).

### Paraffin immunofluorescence

FFPE tissue sections were deparaffinized, rehydrated, and treated with methanol containing 3% hydrogen peroxide to block endogenous peroxidases. Antigen retrieval was performed using citrate buffer (pH 6.0) and microwave heating at medium power for 15 minutes. The sections were blocked with 5% bovine serum albumin (BSA). Two sequential rounds of staining were performed; each included antigen retrieval, blocking, primary antibody incubation, HRP-linked secondary antibody incubation, and tyramide-based signal amplification (TSA). Between staining rounds, antibody removal was accomplished with citrate buffer (pH 6.0) and microwave heating at medium power for 15 minutes. The primary antibodies used were anti-POSTN (ab219056, 1:500) and anti-Integrin β5 (CST, #3629, 1:500). To detect immunofluorescent signals, the PANO IHC kit (10004100100, Panovue, Beijing, China) was used with PPD520-labeled and PPD620-labeled tyramide. Cell nuclei were counterstained with DAPI.

### Pseudobulk differential expression and pathway enrichment analysis

Single-cell transcriptomics enables the evaluation of changes in cell-type-specific gene expression. To assess the effect of CAF-derived POSTN levels on differentially expressed genes (DEGs) in ductal epithelial cells within the CRA001160 (scRNA-seq) dataset, POSTN expression was summed across fibroblasts for each tumor sample. Samples were stratified into high and low POSTN groups using the 75th percentile as a cutoff, and DEGs between these groups were identified.

To this end, ductal epithelial scRNA-seq data were aggregated into a pseudobulk matrix by summing raw gene counts for each sample. Genes with total count ≤1 or detected in fewer than two samples were excluded. DESeq2 (v1.38.3) was used to identify DEGs with |log2 (T/N fold change)| ≥ 1.0 and *P* < 0.05. Gene Set Enrichment Analysis (GSEA) of upregulated genes was annotated using Kyoto Encyclopedia of Genes and Genomes (KEGG) pathways (MSigDB, c2, curated gene sets) and GO pathways (MSigDB, c5, ontology gene sets).

### Correlation analysis of gene expression in bulk RNA-seq data

To validate gene expression correlations in the TCGA-PAAD dataset, data were obtained as described above. The R package corrplot (v0.9.2) was used to calculate and visualize the correlations among the target genes' mRNA expression levels.

### Statistical analysis

Statistical analyses were performed using SPSS 29.0 and GraphPad Prism 10.0. Continuous data are presented as mean ± SD, and categorical data as counts and percentages. Continuous variables were compared using Student's t-tests, Mann-Whitney U tests, or one-way ANOVA, while categorical variables were compared using the chi-square test. Non-normally distributed continuous variables were analyzed using Spearman's correlation. Kaplan-Meier survival analysis was performed using the log-rank test, and independent prognostic factors for OS were determined using the Cox proportional hazards model. Statistical significance was defined as *P* < 0.05 (*P < 0.05, **P < 0.01, ***P < 0.001 and ****P < 0.0001).

## Results

### Integrated single-cell and spatial transcriptomic atlas of PDAC highlights the potential role of fibroblasts in tumor progression

**Figure 1:**
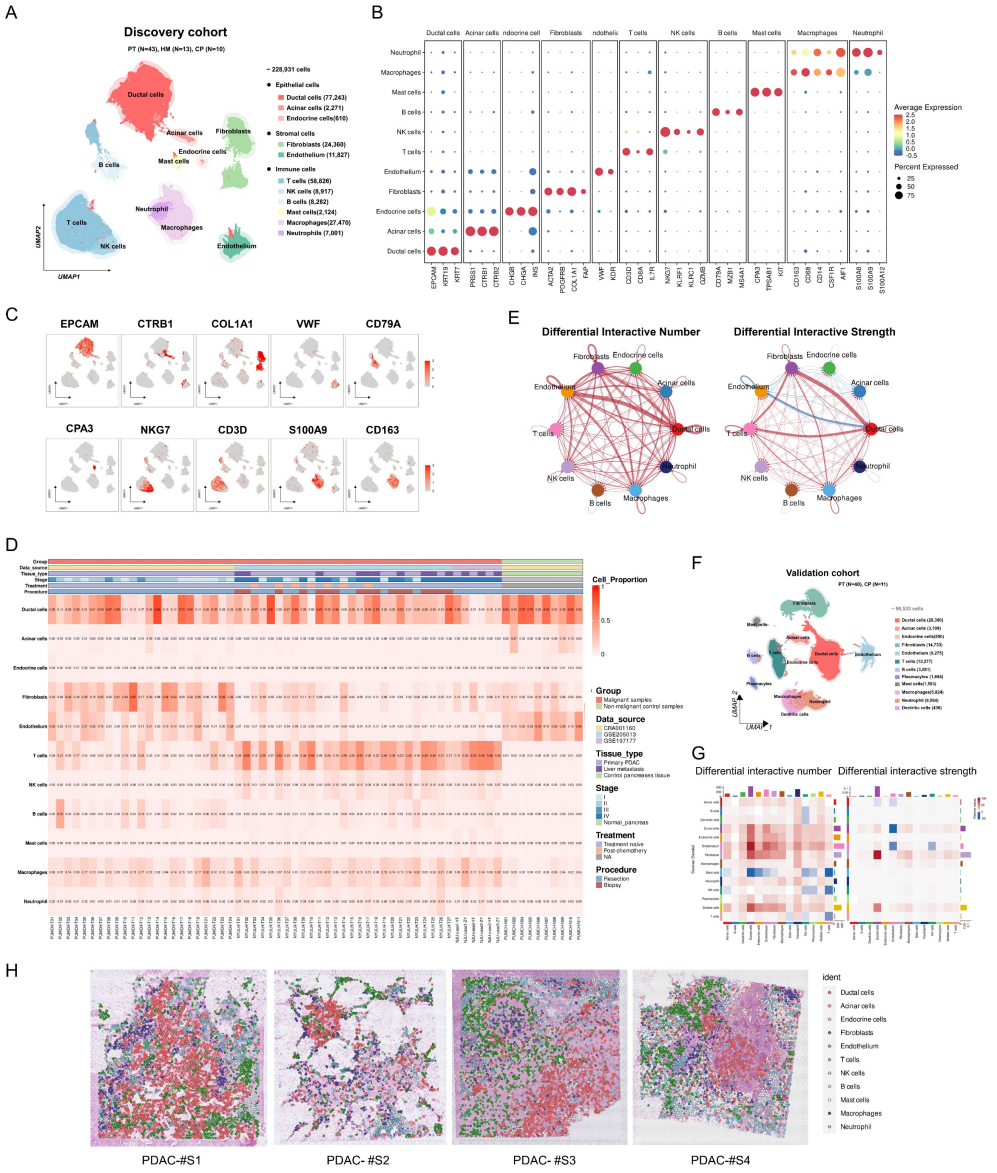
** Integrated single-cell and spatial transcriptomic atlas of PDAC samples. (A)** UMAP plot showing 11 major cell types in the discovery cohort. **(B)** Dot plot illustrating the expression of classical marker genes in major cell types. **(C)** Feature plot showing the expression of classical marker genes in corresponding cell types, with color intensity indicating the expression levels. **(D)** Heatmap showing the proportion of major cell types in each individual sample of the discovery cohort. **(E)** Circle plots showing differential interaction numbers and strengths between malignant and non-malignant samples in the discovery cohort. Blue lines indicate decreased communication, while red lines indicate increased communication in malignant compared to non-malignant pancreatic samples. **(F)** UMAP plot showing major cell types in validation cohort. **(G)** Heatmaps showing differential interaction numbers and strengths between malignant and non-malignant samples in the validation cohort. **(H)** The spatial distribution of major cell types in spatial transcriptome data (CellTrek deconvolution).

To explore the heterogeneity of PDAC at a single-cell resolution, we integrated 66 samples from three single-cell RNA sequencing (scRNA-seq) datasets: CRA001160, GSE205013, and GSE197177 (discovery cohort) (Table S1). This cohort comprised 43 primary PDAC (PT), 13 liver metastasis (LM), and 10 control pancreatic tissue (CP) samples. Overall, a total of 228,931 cells were retained for subsequent analyses and clustered into 11 major cell types (Figure 1A; Figure S1): ductal cells (*EPCAM*, *KRT19*, *KRT7*), acinar cells (*PRSS1*, *CTRB1*, *CTRB2*), endocrine cells (*CHGB*, *CHGA*, *INS*), fibroblasts (*ACTA2*, *PDGFRB*, *COL1A1*, *FAP*), endothelial cells (*VWF*, *KDR*), T cells (*CD3D*, *CD8A*, *IL7R*), NK cells (*NKG7*, *KLRF1*), B cells (*CD79A*, *MZB1*, *MS4A1*), mast cells (*CPA3*, *TPSAB1*, *KIT*), macrophages (*CD163*, *CD68*, *CSF1R*), and neutrophils (*S100A8*, *S100A9*, *S100A12*) (Figure 1B, C)*.* Among these major cell types, ductal cells and fibroblasts exhibited the highest infiltration rates and variability (Figure 1D; Table S6). Furthermore, PDAC samples demonstrated significantly increased numbers and strengths of fibroblast-ductal cell interactions compared to non-malignant samples (Figure 1E). To validate these findings, we included a validation cohort of 50 single-cell transcriptomic samples, with 90,533 cells retained after quality control (Figure 1F; Table S2; Figure S2). This analysis confirmed a pronounced increase in fibroblast-ductal cell interactions in the PDAC samples (Figure 1G). Moreover, spatial deconvolution analysis of four PDAC spatial transcriptomics samples using CellTrek revealed that fibroblasts were predominantly localized adjacent to ductal cells (Figure 1H). These findings indicated that fibroblasts are the predominant cell type interacting with epithelial cells, highlighting the importance of exploring their heterogeneity and interaction profiles with epithelial cells in PDAC.

### Identification of eight fibroblast subtypes within PDAC

**Figure 2:**
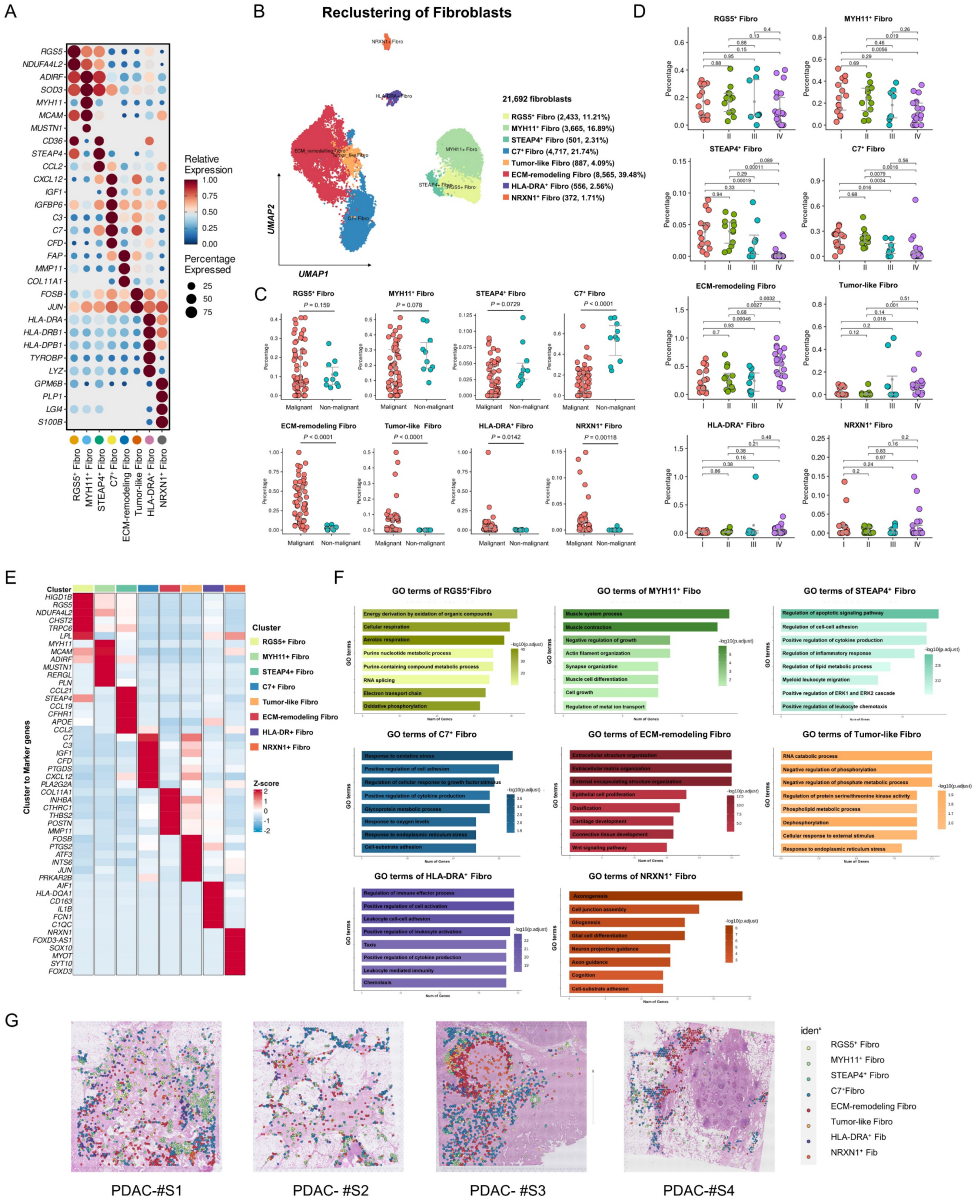
** Heterogeneity analysis of fibroblasts in discovery cohort. (A)** Dot plot showing classical marker gene expression for fibroblast subclusters. **(B)** UMAP plot showing the identified fibroblast subtypes. **(C-D)** Scatterplots depicting the proportions of fibroblast subtypes infiltration in malignant and non-malignant samples, as well as across different clinical stages of PDAC samples in the discovery cohort. *P*-values for group comparisons were calculated using t-tests. **(E)** Heatmap showing z-score normalized expression of canonical markers across fibroblast subtypes. (D) GO analysis of upregulated genes in fibroblast subtypes, with color intensity indicating the scaled -log10 *P*-value. **(F)** The spatial distribution of fibroblast subtypes in PDAC samples (CellTrek).

To elucidate the pivotal role of fibroblasts in shaping the PDAC microenvironment, we utilized previously reported fibroblast subtype-specific markers 4, 8, 21 and categorized 21,629 fibroblasts into eight clusters: RGS5^+^ fibroblasts, MYH11^+^ fibroblasts, STEAP4^+^ fibroblasts, C7^+^ fibroblasts, ECM-remodeling fibroblasts, Tumor-like fibroblasts, HLA-DRA^+^ fibroblasts and NRXN1^+^ fibroblasts (Figure 2A-B, Figure S3). Analysis of infiltration differences among fibroblast subtypes in PDAC samples and non-malignant samples revealed that ECM-remodeling fibroblasts were significantly more abundant in tumors than in non-malignant tissues (Figure 2C). Notably, the proportion of infiltrating ECM-remodeling fibroblasts increased with tumor stage (Figure 2D). To further investigate the relevance of the identified fibroblast subtypes to the prognosis of PDAC patients, TCGA-PAAD bulk RNA-seq data were analyzed using the CIBERSORTx algorithm. High infiltration of ECM-remodeling fibroblasts was significantly associated with poorer overall survival in PDAC patients (*P* = 0.049, Figure S4).

To characterize the expression profiles and functional heterogeneity of diverse fibroblast subtypes, marker genes for each subtype were identified via differential gene expression analysis, followed by GO enrichment analysis of significantly upregulated marker genes (Figure 2E-F, Table S7, Figure S5). RGS5^+^ fibroblasts which exhibit high levels of *RGS5* and *NDUFA4L2*, were notably enriched in cellular respiration and energy metabolism pathways, and resembled the functional profile of normal pancreatic stellate cells 8. MYH11^+^ fibroblasts were enriched in muscle contraction pathways and expressed elevated levels of *ADIRF*, *MCAM*, and *MYH11*, similar to smooth muscle cells 8. STEAP4^+^ fibroblasts, with high levels of *APOE* and *CCL21* expression, were enriched in pathways for cytokine secretion, lipid metabolism, and myeloid cell migration, indicating a transitional state between lipid-processing and inflammatory secretory phenotypes, similar to lipid-processing CAFs (lpCAFs) 21, 22. C7^+^ fibroblasts, with high levels of *C7* and *CXCL12*, were enriched in oxidative stress, growth factor response, and cytokine secretion pathways, representing classic inflammatory CAFs 23. ECM-remodeling fibroblasts were significantly enriched in the extracellular matrix and collagen fibril organization pathways, expressing high levels of *COL1A1*, *MMP11*, and *POSTN*. GSVA analysis of the validation cohort revealed their significant activity in TGF-β1 signaling, EMT, and protein secretion pathways (Figure S3D). Tumor-like fibroblasts exhibited elevated expression of stress-related genes *ATF3* and *JUN*, which were enriched in the stress response and proliferation pathways 24. HLA-DRA^+^ fibroblasts are involved in antigen processing and presentation, aligning with previously identified antigen-presenting CAFs (apCAFs) 25. NRXN1^+^ fibroblasts were enriched in the axonogenesis pathway, with high expression of *NRXN1* and *SOX10*^4^.

### Cell-state evolutionary trajectory and transcriptional regulatory features of different fibroblast subpopulations

**Figure 3:**
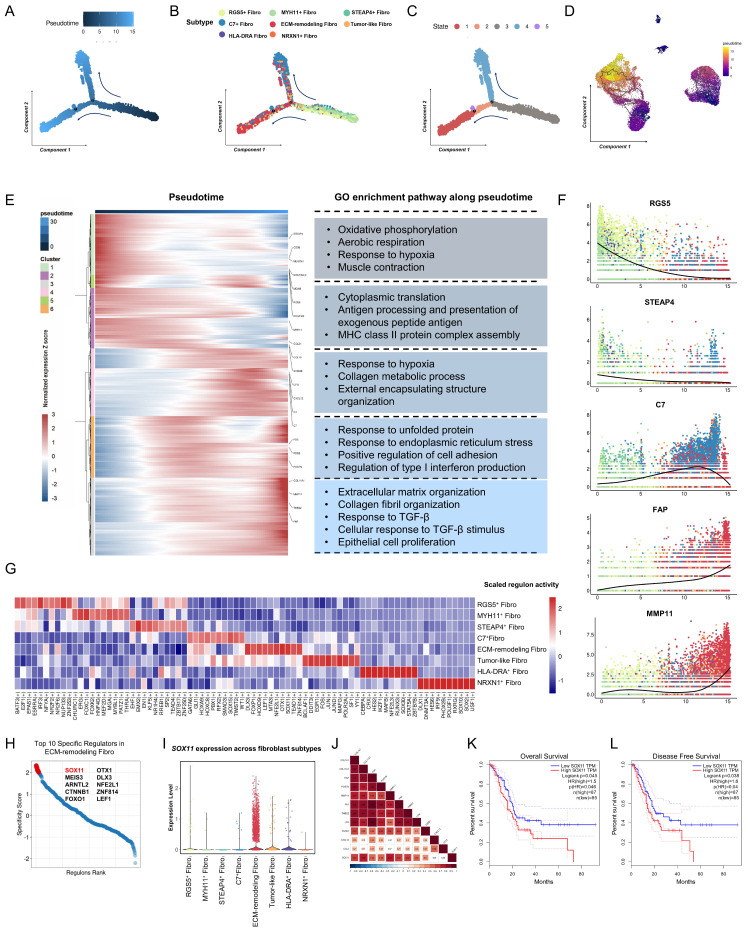
** Evolutionary trajectories and transcriptional regulatory analysis of fibroblast subtypes in the discovery cohort. (A)** Pseudotime analysis of fibroblast developmental trajectories inferred by Monocle2, with blue intensity indicating the temporal order (dark blue representing the starting state). **(B-C)** Evolutionary trajectories of fibroblasts inferred by Monocle2, colored by subtype (B) and state (C). **(D)** Validation of fibroblast evolutionary trajectories using Monocle3. **(E)** Heatmap showing scaled expression of differentially expressed genes along the pseudotime trajectory. Bar plots on the right highlight the top significantly enriched pathways for each gene cluster. **(F)** Pseudotime projection of fibroblast subtype marker expression, illustrating the developmental trajectories of fibroblast subtypes in PDAC samples. **(G)** Heatmap showing the mean activity of top differentially activated regulons in each fibroblast subtype, inferred by pySCENIC. **(H)** Dot plot ranking the top differentially activated regulons in ECM-remodeling fibroblasts based on regulon-specific scores. **(I)** Violin plot showing *SOX11* expression across fibroblast subtypes. **(J)** Triangle heatmap of Pearson correlations between *SOX11* mRNA expression and marker gene of ECM-remodeling fibroblasts expression in the TCGA-PAAD dataset. **(K-L)** Kaplan-Meier curves for overall survival (K) and disease-free survival (L) of TCGA-PAAD patients stratified by high- and low- *SOX11* mRNA expression levels.

To investigate the dynamic processes of fibroblast subtypes at single-cell resolution, pseudotime trajectory analysis was performed. The results identified two major trajectories: RGS5^+^ fibroblasts and MYH11^+^ fibroblasts acted as progenitors, whereas STEAP4^+^ fibroblasts and Tumor-like fibroblasts represented transitional states, terminally evolving into ECM-remodeling fibroblasts or C7^+^ fibroblasts (Figure 3A-D, Figure S6A-C). Gene expression patterns during state transitions were analyzed and presented in a smoothed heatmap (Figure 3E, Figure S6D). Early-stage genes were enriched in pathways associated with aerobic respiration, muscle contraction, and oxidative phosphorylation. As pseudotime progressed, gene expression shifted towards pathways related to hypoxia response, collagen metabolism, and the unfolded protein response, ultimately highlighting tumor progression pathways such as ECM organization and TGF-β signaling. Specifically, the expression of *RGS5* decreased over pseudotime, while *STEAP4* was expressed in both initial and inflammatory states. *C7* was predominantly expressed at the terminal stage. Moreover, ECM-related genes, such as *FAP* and *MMP11*, were highly expressed at the terminal stage (Figure 3F).

**Figure 4:**
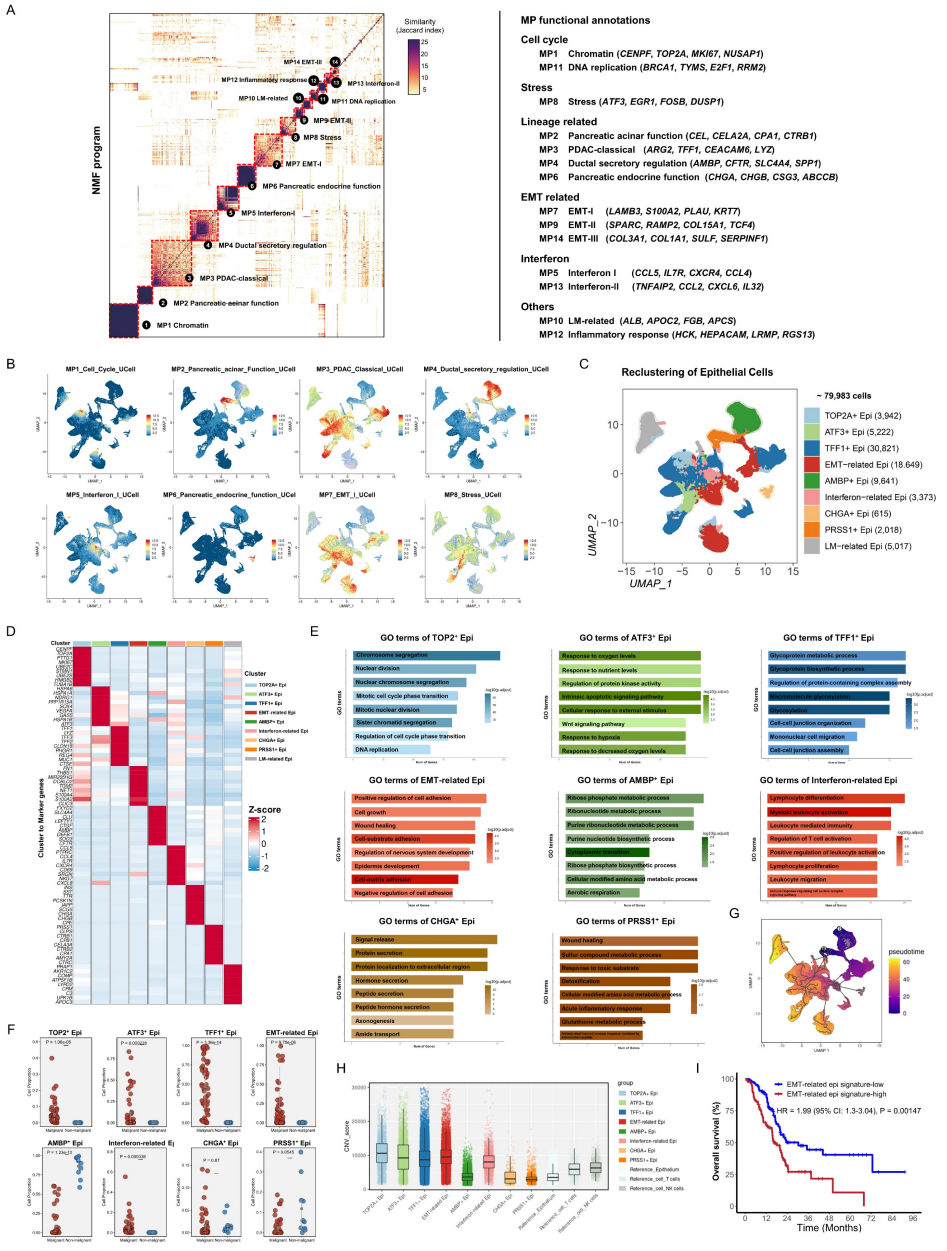
** Identification of epithelial meta-programs in the discovery cohort. (A)** Heatmap showing Jaccard similarity indices for robust NMF programs based on the top 50 genes. Programs are grouped into meta-programs (MPs, red dashed lines), with MP families labeled on the right. **(B)** Feature plot of epithelial meta-program gene list scores calculated using the AddModuleScore function in Seurat (MP1-MP8). Colors indicate score levels, ranging from no expression (blue) to high expression (red). **(C)** UMAP plot visualizing epithelial subclusters, with distinct colors representing different subtypes. **(D)** Scatterplot of epithelial subtype infiltration proportions in malignant *vs.* non-malignant samples. P-values were calculated using t-tests for group comparisons. **(E)** Heatmap showing z-score normalized expression of canonical markers across epithelial subtypes.** (F)** GO enrichment analysis of upregulated genes significantly enriched in each epithelial subtype. **(G)** CNV score levels of epithelial subtypes inferred by inferCNV. **(H)** Kaplan Meier curves for OS of TCGA-PAAD patients stratified by EMT-related epithelial subtype-specific signature expression.

To elucidate fibroblast plasticity from a single-cell perspective, pySCENIC analysis was performed to identify activated transcription factors (TFs) within fibroblast subtypes, uncovering distinct TF activity profiles (Figure 3G, Table S8, Figure S6E). Notably, *SOX11* exhibited enhanced expression and activity within the regulatory network of ECM-remodeling fibroblasts (Figure 3G). The Regulon-specific score (RSS) analysis identified *SOX11* as a top-specific TF in ECM-remodeling fibroblasts (Figure 3H, Figure S6F). *SOX11* was predominantly expressed in fibroblasts, particularly in the ECM-remodeling subpopulation (Figure 3I, Figure S7). Analysis of the TCGA-PAAD RNA-seq dataset revealed a significant correlation between *SOX11* expression and ECM-remodeling fibroblast markers, including *COL11A1*, *FAP*, *MMP11*, *POSTN*, and *THBS2* (Figure 4K). High *SOX11* mRNA expression was associated with shorter OS (Log-rank *P* = 0.045) and disease-free survival (DFS) (Log-rank *P* = 0.038) (Figure 4J-L). These findings suggested that SOX11 may function as a master regulator in promoting fibroblast differentiation toward an ECM-remodeling phenotype in PDAC.

### Identification of epithelial meta-programs reveals heterogeneity in PDAC

The significant cellular communication between fibroblasts and epithelial cells observed in both the discovery and validation cohorts prompted further investigation of subpopulation interactions, highlighting the importance of understanding epithelial cell heterogeneity in PDAC. In the discovery cohort, 79,983 epithelial cells were analyzed using a proven NMF-based method to identify co-expressed gene programs 19, 26. NMF (*K* = 3-10) was employed to analyze epithelial cell expression matrices across all samples. This approach decomposed the original expression matrix into several non-negative sub-matrices, each defined by their top 50 expressed genes. Through this analysis, 3,432 expression programs were extracted from 66 samples. Subsequently, reliable and non-redundant expression programs were selected based on their intra- and inter-sample similarities. These programs were then hierarchically clustered using the Jaccard similarity index, resulting in 14 recurrent, stable expression patterns across samples, defined as "Meta-programs" (MPs) (Figure 4A, Table S9). GO enrichment analysis of the 50 genes in each MP enabled the annotation and classification of these 14 MPs into six functional families (Table S10; Figure 4A, Right), including generic programs (cell cycle, stress response, EMT, and interferon signaling) and lineage-specific programs. Notably, MP2, MP4, and MP6 were associated with normal pancreatic functions, whereas MP3 represented the PDAC-classical program 19.

Based on the MP analysis, epithelial cells were further subclustered, revealing nine distinct epithelial cell states within the discovery cohort: TOP2A^+^ epithelial cells, ATF3^+^ epithelial cells, TFF1^+^ epithelial cells, EMT-related epithelial cells, AMBP^+^ epithelial cells, Interferon-related epithelial cells, CHGA^+^ epithelial cells, PRSS1^+^ epithelial cells, and liver metastasis (LM)-related epithelial cells (Figure 4B-C, Figure S8). Functional characteristics were inferred from marker genes, GO analyses, and infiltration proportions (Figure 4D-F, Table S11). AMBP^+^ epithelial cells exhibited high expression of *FXYD2*, *SCLA4A*, and *AMBP*, which were enriched in energy metabolism, and predominated in non-malignant samples, suggesting premalignant ductal-like roles 7, 16. TOP2A^+^ epithelial cells are primarily enriched in the cell cycle and DNA replication pathways, indicating proliferative cancer cells 14. ATF3^+^ epithelial cells were enriched in pathways related to response to oxygen levels and regulation of protein kinase activity, indicating stress conditions in tumor cells. TFF1^+^ epithelial cells, with high levels of *TFF1*, *LYZ*, *CLDN18*, and *MUC1*, were aligned with the PDAC-classical subtype and were primarily enriched in pathways related to glycoprotein metabolism 19, 26, 27. EMT-related epithelial cells, characterized by the expression of genes such as *FN1* and *VIM*, showed activation of pathways that regulate cell adhesion, wound healing, and cell growth, suggesting their potential role in tumor invasion and metastasis.

Interferon-related epithelial cells expressed genes like *CCL5* and *CXCR4*, which are involved in myeloid leukocyte activation, T-cell regulation, and leukocyte-mediated immunity, potentially suggesting a role in recruiting or activating immune cells by tumor cells 26, 28. CHGA^+^ epithelial cells, which perform acinar functions, expressed high levels of *INS* and *SST* and were enriched in pathways for protein secretion and extracellular region localization 7. PRSS1^+^ epithelial cells, characterized by *CTRB1* and *CPB1* expression, were involved in sulfur compound metabolism and pancreatic endocrine functions. LM-related epithelial cells, derived from liver metastasis, exhibited no distinct pathway enrichment, suggesting hepatocyte contamination, and were excluded from subsequent analyses.

Pseudotime trajectory analysis was performed to explore the dynamic changes and transcriptional state evolution of epithelial subpopulations in PDAC tissue. AMBP^+^ epithelial cells, predominantly enriched in control pancreatic tissues and representing near-normal ductal epithelial cells, and PRSS1^+^ epithelial cells, representing pancreatic acinar epithelial cells, were selected as potential progenitors at the trajectory start. The results revealed that TFF1^+^ epithelial cells, EMT-related epithelial cells, ATF3^+^ epithelial cells, and TOP2A^+^ epithelial cells were identified at transitional or terminal stages (Figure 4G). To assess malignancy, inferCNV analysis revealed significantly higher CNV scores in TFF1^+^ epithelial cells, EMT-related epithelial cells, ATF3^+^ epithelial cells, and TOP2A^+^ epithelial cells than in the reference cells, confirming their malignant nature (Figure 4H). Validation using the TCGA-PAAD RNA-seq cohort indicated that higher signature scores for EMT-related epithelial cells (Log-rank *P* = 0.00147) was significantly associated with worse overall survival (Figure 4I). This suggests that EMT-related epithelial cells are terminally differentiated malignant cells associated with poor clinical outcomes.

### ECM-remodeling fibroblast-derived POSTN facilitates stroma-tumor communication and its expression correlates with tumor malignancy and prognosis of PDAC

**Figure 5:**
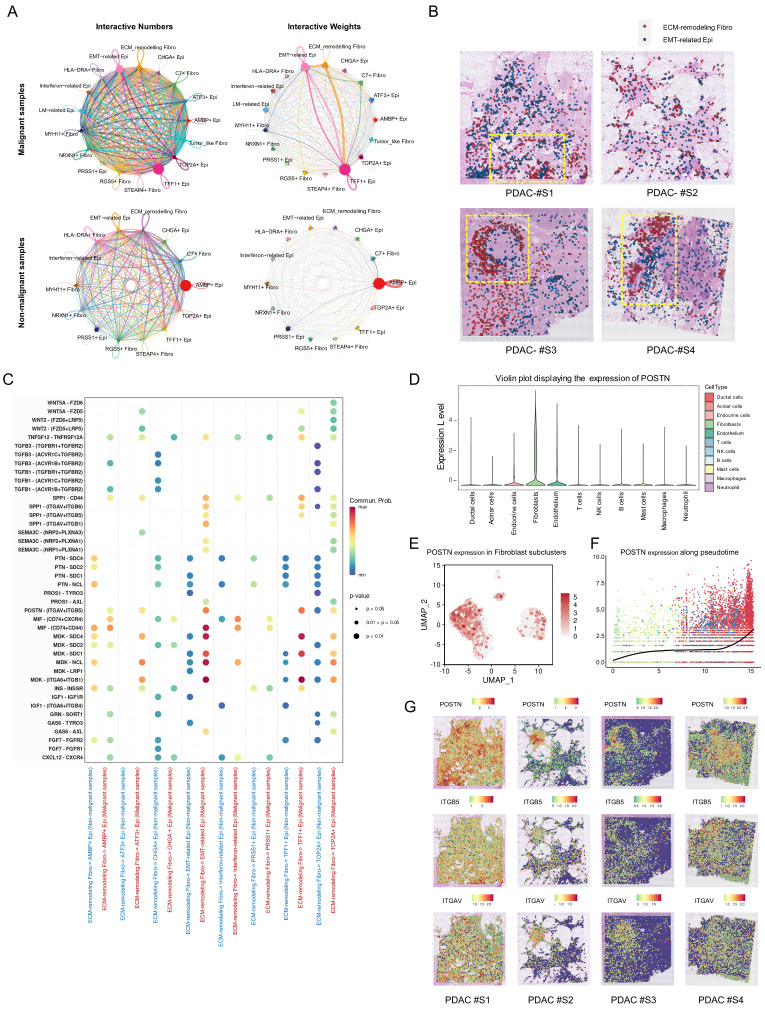
** Potential interactions between ECM-remodeling fibroblasts and the EMT-related epithelial subtype with POSTN as a key secreted ligand. (A)** Circle plots comparing cell-cell communication networks, showing the number and strength of interactions between fibroblast and epithelial subpopulations in malignant versus non-malignant samples. **(B)** Spatial distribution of ECM-remodeling fibroblasts and EMT-related epithelial cells in PDAC samples based on CellTrek deconvolution, with yellow boxes indicating potential co-localization regions. **(C)** Bubble plots comparing secreted ligand-receptor pairs from ECM-remodeling fibroblasts targeting epithelial subtypes between malignant and non-malignant samples. **(D)** Violin plot showing *POSTN* expression across all samples in the discovery cohort, grouped by major cell types. **(E)** Feature plot highlighting predominant *POSTN* expression in the ECM-remodeling fibroblast subpopulation.** (F)** Pseudotime projection plot showing gradual upregulation of *POSTN* along the fibroblast evolutionary trajectory. **(G)** Spatial feature plot showing the expression of *POSTN*, *ITGAV*, and *ITGB5*.

Following the identification of fibroblasts and epithelial heterogeneity in PDAC samples, CellChat was used to assess the interactions among the subpopulations. Malignant samples exhibited enhanced interactions between subgroups compared to non-malignant samples, particularly strong signals from ECM-remodeling fibroblasts to EMT-related epithelial cells (Figure 5A). Acknowledging that spatial proximity implies potential interactions, we analyzed co-localization in spatial samples. CellTrek deconvolution analysis identified the frequent co-localization of ECM-remodeling fibroblasts and EMT-related epithelial cells (Figure 5B). To further investigate ligand-receptor interactions, we found that ECM-remodeling fibroblasts in malignant samples secreted soluble ligands, including *POSTN*, *MDK*, and *MIF*, which were significantly upregulated compared with those in non-malignant samples and acted as molecular messengers to epithelial subtypes (Figure 5C). *POSTN* was predominantly expressed by fibroblasts (Figure 5D, Figure S9A-B), especially within the ECM-remodeling subtype (Figure 5E), with its expression increasing along the pseudotime trajectory and peaking at the terminal stage (Figure 5F). As *SOX11* has been identified as a potential key TF regulating ECM-remodeling fibroblasts, we grouped fibroblasts by *SOX11* expression, revealing that *POSTN* was among the most upregulated genes in the *SOX11*-high group (Figure S9C, Table S12). A significant correlation between *POSTN* and *SOX11* mRNA expression was also observed in the TCGA-PAAD RNA-seq dataset (Spearman's *R* = 0.62, *P* < 0.0001) (Figure S9D). Furthermore, SOX11 overexpression in primary CAFs significantly upregulated POSTN expression (Figure S9E). Spatial characterization revealed co-localization with its receptors *ITGAV* and *ITGB5* (Figure 5G). Based on these findings and the existing literature 5, 29, 30, we propose that POSTN-ITGAV/ITGB5 may act as a key ligand-receptor pair driving ECM-remodeling fibroblasts to promote PDAC progression.

**Figure 6:**
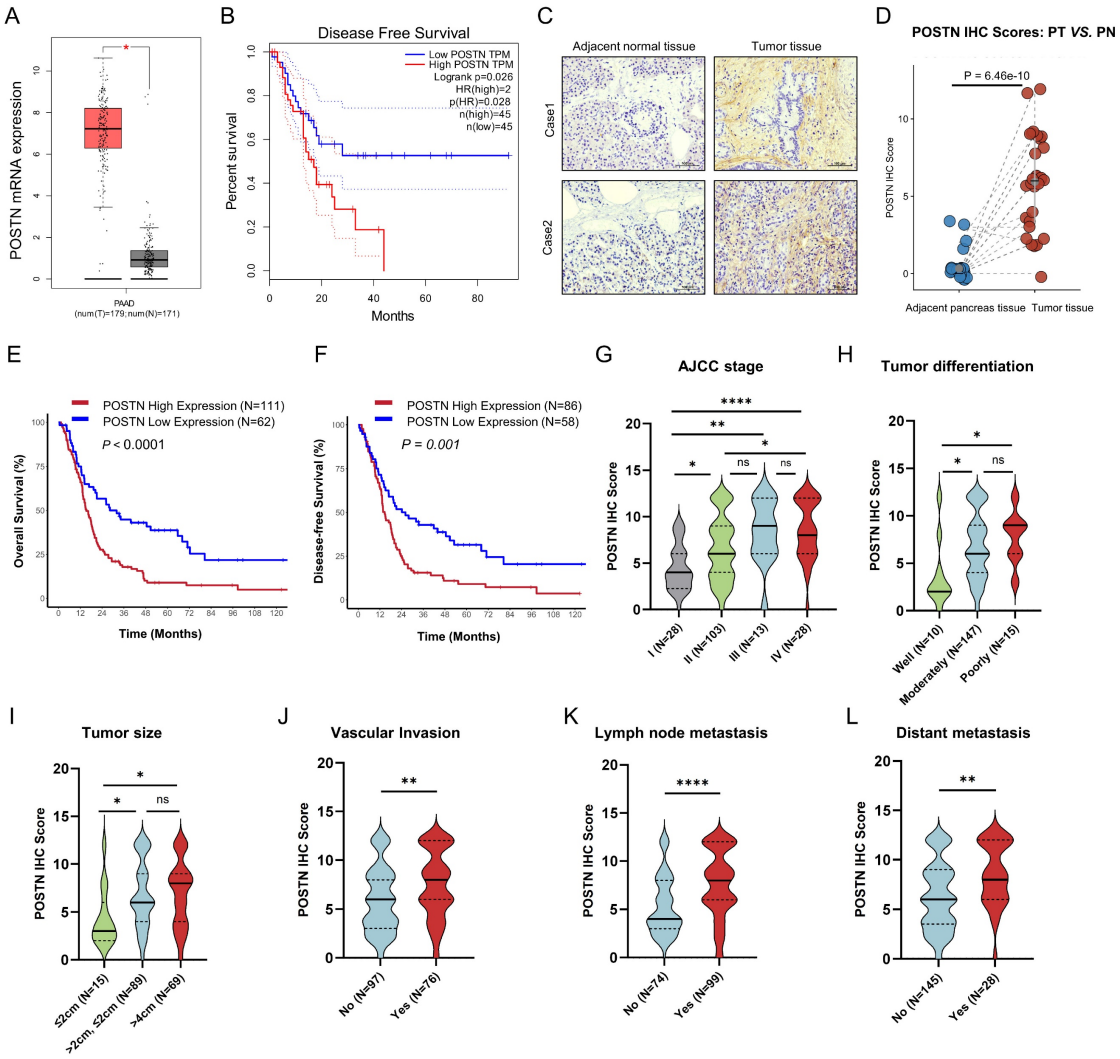
** POSTN is highly expressed in the stromal area of PDAC and is positively associated with tumor progression and overall survival. (A)** POSTN mRNA levels in pancreatic cancer tissues (*n* = 179) compared to normal tissues (*n* = 171, GTEx matched) analyzed via the GEPIA database. **(B)** Kaplan Meier curves for DFS of TCGA-PAAD patients stratified by high- and low- *POSTN* mRNA expression levels (cutoff: quantile) via GEPIA. **(C)** Representative IHC staining images of POSTN in PDAC tissues and paired adjacent normal tissues from patients in the PUCH-PDAC cohort. **(D)** IHC scores of POSTN in 30 paired PDAC and adjacent normal tissues (*n* = 30 per group). **(E-F)** Kaplan Meier curves for OS (E) and DFS (F) of patients within PUCH-PDAC cohort stratified by high- and low- POSTN expression levels. **(G-L)** Associations between POSTN IHC scores and clinical features, including TNM stages, tumor size, vascular invasion, tumor differentiation, lymph node metastasis, and distant metastasis.

To further characterize POSTN expression in PDAC, we first confirmed its mRNA levels using the TCGA-PAAD RNA-seq dataset via GEPIA (http://gepia.cancer-pku.cn/) 20, which revealed significantly higher expression in PDAC tissues than in normal tissues (Figure 6A). Additionally, higher *POSTN* mRNA levels, defined by the 75th percentile cutoff, were significantly associated with reduced DFS (Log-rank *P* = 0.026) (Figure 6B). In our PUCH-PDAC cohort, we determined the clinical significance and prognostic value of POSTN expression in patients with PDAC. Using IHC, we analyzed 173 patients, including 30 paired tumor and adjacent tissue samples. Based on our IHC scoring standard, POSTN protein expression increased markedly in tumor tissues compared with that in adjacent normal tissues (*P* < 0.001), consistent with the scRNA-seq results (Figure 6C, D).

Kaplan-Meier analysis of OS and DFS revealed that patients with high POSTN expression levels had unfavorable OS (Log-rank *P* < 0.0001) and DFS (Log-rank *P* = 0.001) (Figure 6E, F). Elevated POSTN expression was observed with advancing tumor stage, poor differentiation, and greater tumor size, and was significantly associated with vascular invasion, lymph node metastasis, and distant metastasis (Figure 6G-L). Patients were categorized into high and low POSTN expression groups, which revealed significant associations with tumor differentiation (*P* = 0.006), TNM stage (*P* < 0.001), tumor size (*P* = 0.006), lymph node metastasis (*P* < 0.001), vascular invasion (*P* = 0.003), and distant metastasis (*P* = 0.002) (Table 1). We assessed the prognostic significance of POSTN expression in patients with PDAC using univariate and multivariate Cox regression analyses (Table 2). Univariate analysis identified POSTN as a significant predictor of OS with an HR of 2.09 (95% CI: 1.44-3.03;* P* < 0.001). In the multivariate analysis, POSTN expression remained an independent predictor of OS, with an HR of 1.71 (95% CI: 1.09-2.68; *P* = 0.019). Our results suggest that POSTN expression is a stromal marker and independent prognostic biomarker in PDAC, highlighting its potential clinical utility for risk assessment and outcome prediction for patients with PDAC.

### CAF-derived POSTN enhances the growth and colony formation of PDAC cells

**Figure 7:**
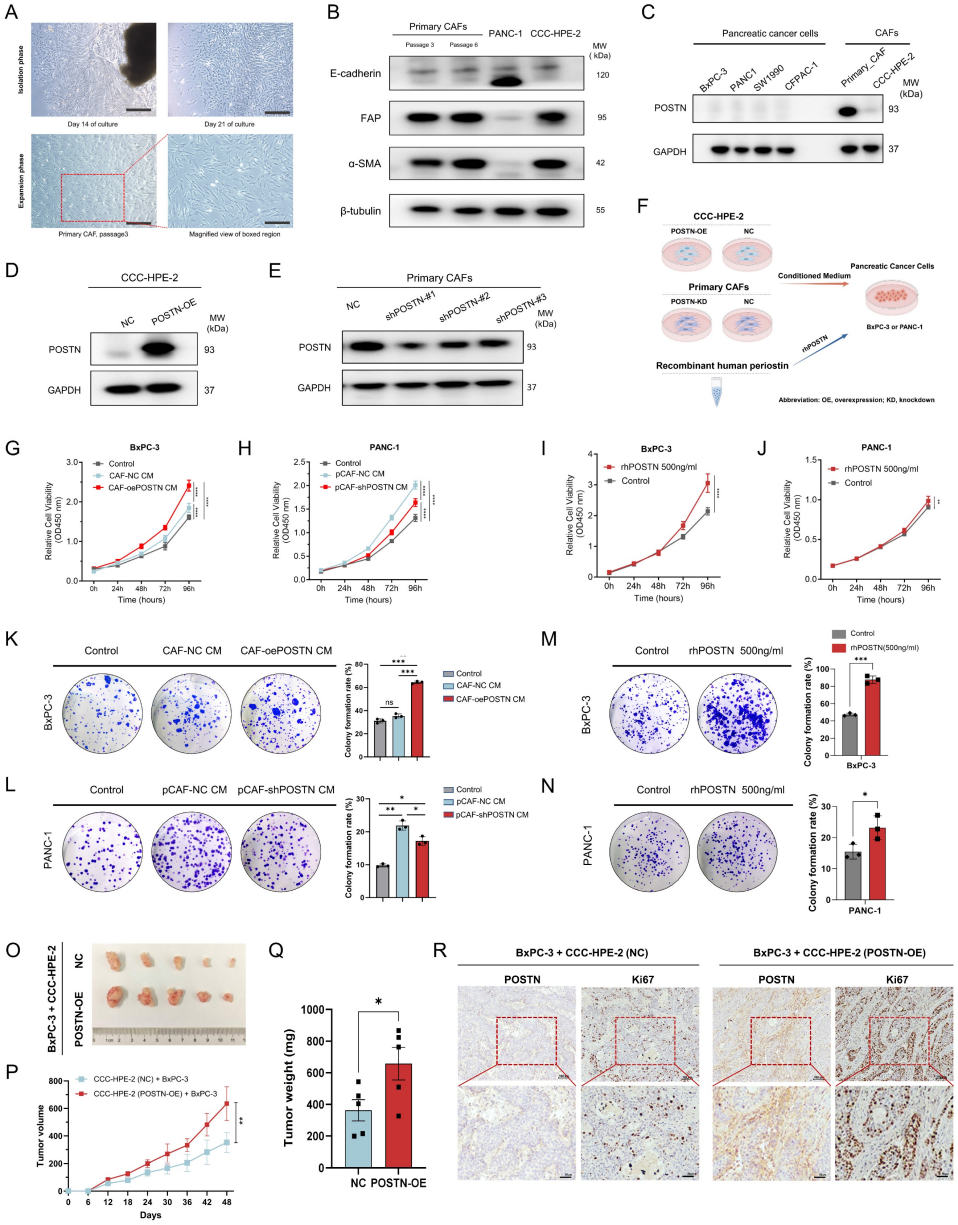
** CAF-derived POSTN promotes PDAC cell growth and colony formation. (A)** Isolation of primary CAF from fresh surgically resected PDAC tissue. Representative images of the primary CAF extraction process at 14 days and 21 days after adherent culture of tissue pieces. **(B)** Characterization of primary CAFs via Western blot showing FAP, α-SMA, and E-cadherin expression. *P3* and *P6* in (A-B) denote the passage numbers of the primary CAFs, corresponding to passages 3 and 6, respectively. **(C)** Western blot analysis of POSTN expression in PDAC cell lines and CAF cells. **(D-E)** Overexpression efficiency of POSTN in the CCC-HPE-2 cell line and knockdown efficiency of POSTN in primary CAFs analyzed by Western blot.** (F)** Schematic illustration of the phenotypic experimental design. **(G-H)** CCK-8 assays assessing BxPC-3 (G) and PANC-1 (H) proliferation after treatment with (1) control medium, (2) 40% CM from CAF-oePOSTN or pCAF-shPOSTN cells, and (3) 40% CM from CAF-NC or pCAF-NC cells. **(I-J)** CCK-8 assays assessing BxPC-3 and PANC-1 proliferation after treatment with rhPOSTN. **(K-L)** Colony formation assays evaluating BxPC-3 and PANC-1 growth under conditions similar to Figures 7G and 7H, respectively. **(M-N)** Colony formation assays assessing BxPC-3 and PANC-1 growth after treatment with rhPOSTN. **(O-Q)** Tumor growth and burden analysis in BALB/c-Nude mice injected subcutaneously with BxPC-3 cells (2 × 10⁶) mixed with CCC-HPE-2 (POSTN-OE) cells (2 × 10⁶) or CCC-HPE-2 (NC) cells (2 × 10⁶). **(R)** Representative IHC staining images of xenograft tumor sections stained for Ki-67 and POSTN.

To explore the role of CAF-derived POSTN in PDAC progression, we first isolated primary CAFs from fresh surgically resected PDAC tissues to establish a PDAC-derived CAF cell (Figure 7A). Western blot confirmed the high expression of the CAF markers FAP and α-SMA, with no detection of the epithelial marker E-cadherin, validating successful CAF isolation (Figure 7B). Additionally, we employed CCC-HPE-2 cells, derived from human embryonic pancreatic fibroblasts 12, as a model of pancreatic CAFs, which also showed high FAP and α-SMA expression. Evaluation of POSTN expression indicated low levels in pancreatic cancer cell lines but markedly higher levels in primary CAFs; and CCC-HPE-2 cells exhibited lower POSTN expression (Figure 7C).

We established CCC-HPE-2 cell lines with stable POSTN overexpression (CAF-oePOSTN) and corresponding control cells (CAF-NC) using lentiviral vectors (Figure 7D). Similarly, primary CAFs with stable POSTN knockdown (pCAF-shPOSTN) and their negative controls (pCAF-NC) were generated (Figure 7E). Their conditioned media (CM) were added to the PDAC cell lines (BxPC-3 or PANC-1) for *in vitro* analysis (Figure 7F). To further confirm the secretion of POSTN in the CAF-tumor coculture system, ELISA assays were performed to quantify POSTN levels in the conditioned media. The results showed a significant increase of POSTN concentration in the conditioned medium from POSTN-overexpressing CCC-HPE-2 cells compared to control cells (Figure S10A). Conversely, a marked decrease in POSTN concentration was observed in the conditioned medium from POSTN-knockdown primary CAF cocultures (Figure S10B). *In vitro* assays demonstrated that the conditioned medium from CCC-HPE-2 cells (CAF-oePOSTN CM) significantly enhanced the proliferation and colony formation of BxPC-3 cells compared to CAF-NC CM (Figure 7G, K). Treatment of BxPC-3 and PANC-1 cells with rhPOSTN produced similar results, confirming these effects (Figure 7I-J, M-N). Additionally, treatment of PANC-1 cells with conditioned medium from primary CAFs with POSTN knockdown (pCAF-shPOSTN CM) significantly reduced cell proliferation and colony formation compared to treatment with medium from control CAFs (pCAF-shNC CM), indicating that POSTN is essential for the tumor-promoting effects of CAFs (Figure 7H, L).

To assess the role of POSTN in tumorigenicity of pancreatic cancer cells *in vivo*, we established a subcutaneous cell-derived xenograft (CDX) model by co-injecting BxPC-3 cells with CAF-NC or CAF-POSTN into BALB/c-Nude mice. Tumors from the BxPC-3 + CCC-HPE-2 (POSTN-OE) co-injections group showed significantly larger volumes and masses than those from the BxPC-3 + CCC-HPE-2 (NC) group (Figure 7O-Q). IHC analysis demonstrated higher levels of POSTN and the proliferation marker Ki-67 in xenografts from the BxPC-3 + CCC-HPE-2 (POSTN-OE) group compared to controls (Figure 7R). These findings highlight the crucial role of CAF-derived POSTN in tumor progression via paracrine signaling.

### CAF-derived POSTN induces the translation to EMT-subtype and invasiveness via PI3K/AKT/β-catenin signaling in PDAC cells

**Figure 8:**
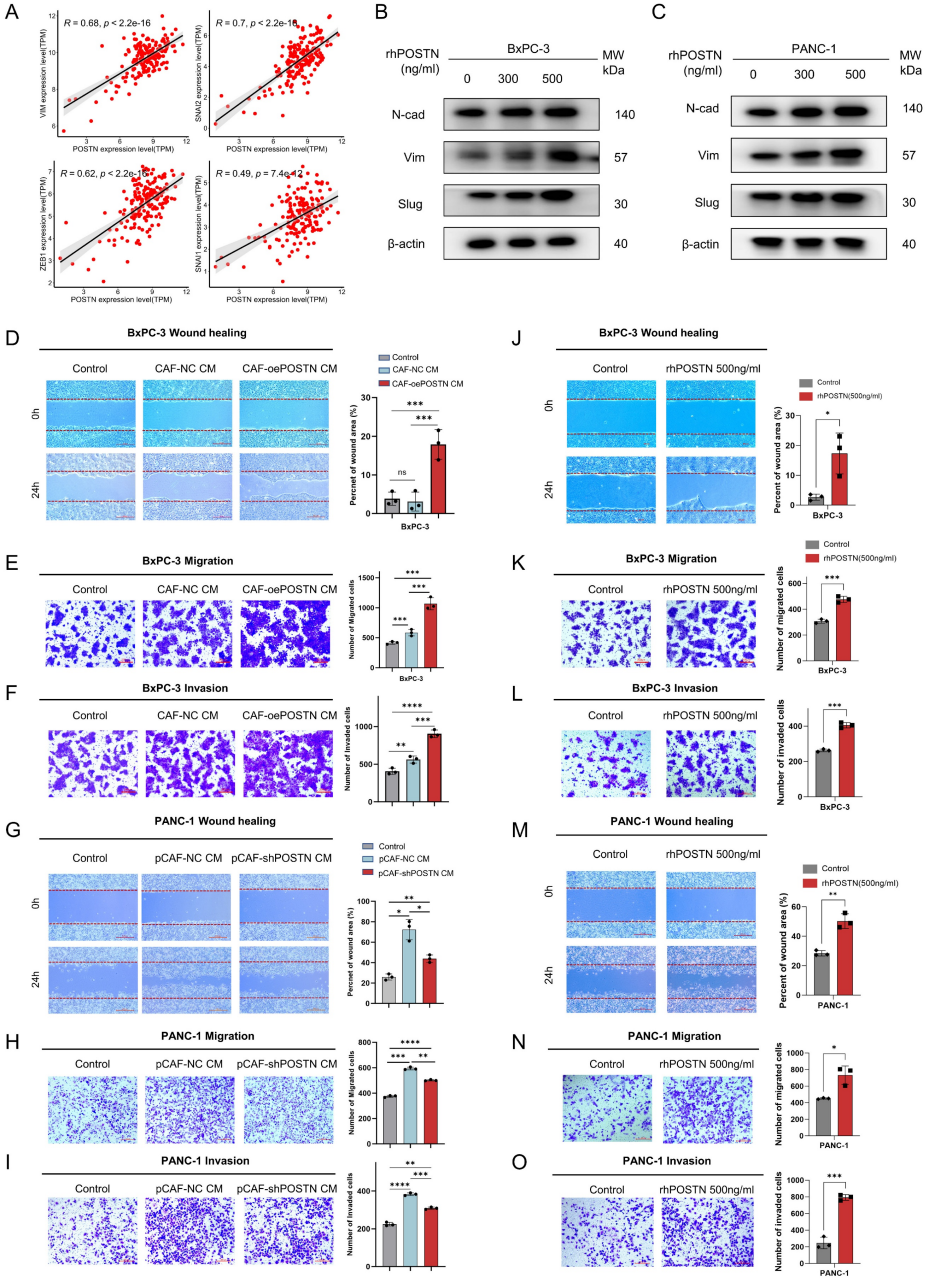
** CAF-derived POSTN induces EMT, migration, and invasion of PDAC cells. (A)** Correlation analysis between *POSTN* mRNA expression and EMT markers (VIM, SNAI1, ZEB1, SNAI2) in the TCGA-PAAD dataset. **(B-C)** Western blot analysis of EMT markers (N-cadherin, vimentin, and slug) in BxPC-3 and PANC-1 cells treated with rhPOSTN at indicated concentrations for 24 hours. **(D-F)** Cellular wound healing, migration, and invasion assays for BxPC-3 cells under conditions similar to Figure 7G. **(G-I)** Cellular wound healing, migration, and invasion assays for PANC-1 cells under conditions similar to Figure 7H. **(J-O)** Wound healing and transwell assays evaluating migration and invasion ability of BxPC-3 and PANC-1 cells treated with varying concentrations of rhPOSTN.

After recognizing the pivotal role of POSTN as a mediator in fibroblast-cancer cell communication, we validated the function of POSTN in promoting EMT in PDAC cells. Analysis of the TCGA-PAAD RNA-seq dataset (*n* = 178) showed strong positive correlations between POSTN and EMT markers such as VIM, SNAI1, SNAI2, and ZEB1 (Figure 8A). Treatment of BxPC-3 and PANC-1 cells with rhPOSTN at 0, 300, and 500 ng/mL for 24 h significantly upregulated EMT markers, such as vimentin, N-cadherin, and slug, suggesting the induction of EMT phenotypic changes (Figure 8B-C). To assess whether POSTN-driven EMT enhances tumor aggressiveness, we evaluated its impact on cell migration and invasion. BxPC-3 cells treated with conditioned medium from CCC-HPE-2 (oePOSTN) cells (CAF-oePOSTN CM) showed significantly enhanced migration and invasion compared with those treated with control CAF-NC CM (Figure 8D-F). Similar enhancement was observed following rhPOSTN treatment of BxPC-3 and PANC-1 cells (Figure 8J-O). Treatment of PANC-1 cells with conditioned medium derived from POSTN-knockdown CAFs (pCAF-shPOSTN CM) resulted in markedly decreased cell migration and invasion relative to treatment with medium from control CAFs (pCAF-NC CM) (Figure 8G-I). These findings suggest that POSTN is a key mediator of fibroblast-cancer cell communication, promoting EMT and subsequent tumor invasiveness.

**Figure 9:**
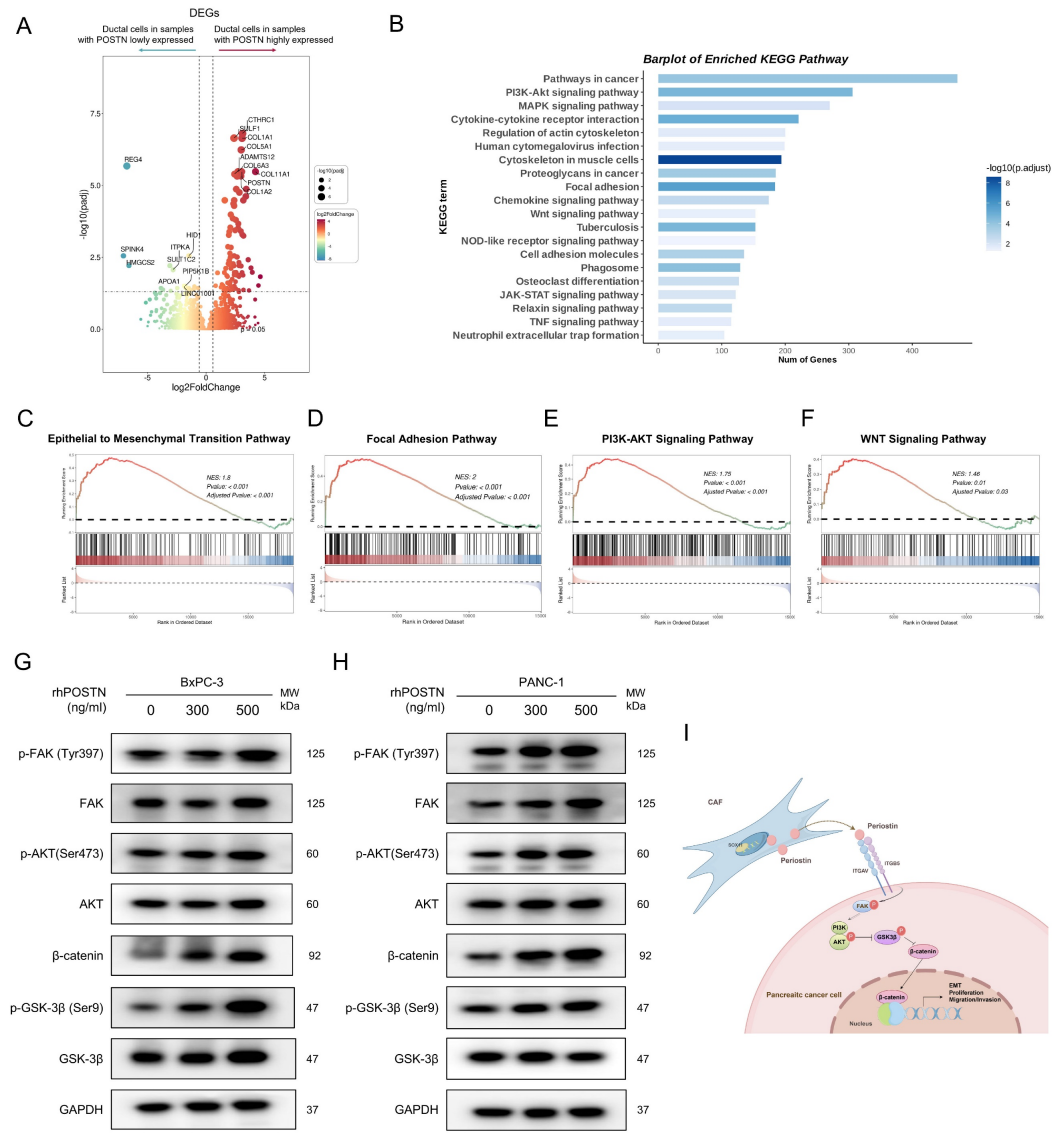
** CAF-derived POSTN induces the translation to EMT-subtype via PI3K/AKT/β-catenin signaling in PDAC Cells. (A)** Differential gene expression analysis of ductal cells based on fibroblast-derived POSTN levels. Tumor samples from the CRA001160 dataset were classified into high- and low- POSTN groups (75th percentile cutoff), and ductal cell gene expression profiles were compared to identify differences linked to CAF-derived POSTN. **(B)** GSEA of upregulated genes in ductal cells from POSTN-high samples identified enriched KEGG pathways. **(C-F)** GSEA of upregulated genes revealed enrichment in the EMT pathway, focal adhesion pathway, PI3K-AKT pathway, and WNT pathway. **(G-H)** Western blot analysis of β-catenin expression, and phosphorylation of FAK, AKT, and GSK-3β in BxPC-3 and PANC-1 cells after 24-hour treatment with rhPOSTN at varying concentrations. **(I)** Schematic illustration of how CAF-derived POSTN drives the EMT phenotype in PDAC cells via integrin αvβ5/FAK/PI3K/AKT/β-catenin signaling pathway.

To further elucidate the molecular mechanisms of POSTN-mediated EMT and tumor aggressiveness, we used scRNA-seq samples from the CRA001160 dataset. Tumor samples were classified into high- and low-*POSTN* expression groups based on the 75th percentile of fibroblast-derived *POSTN* expression. We analyzed differential gene expression in the ductal cells between the two groups to assess expression profile differences linked to varying CAF-derived POSTN levels (Figure 9A, Table S13). GSEA analyses of the upregulated genes in ductal cells from high-*POSTN* samples revealed significant enrichment in the EMT, focal adhesion, PI3K-AKT, and WNT signaling pathways (Figure 9B-F). Experimental validation in BxPC-3 and PANC-1 cells treated with rhPOSTN showed increased β-catenin levels and enhanced phosphorylation of FAK, AKT, and GSK-3β (Figure 9G-H), confirming the role of POSTN in activating the PI3K/AKT/β-catenin signaling axis in promoting PDAC progression (Figure 9I).

To extend these observations *in vivo*, immunohistochemistry (IHC) was performed on tumor tissues from the CDX model to assess the activation of downstream signaling pathways regulated by POSTN. IHC results showed that integrin β5, the predicted receptor for POSTN, was expressed in both POSTN-overexpressing group and control group. However, the downstream effector β-catenin and EMT markers (Slug, N-cadherin) exhibited significantly higher expression in xenografts from the BxPC-3 + CCC-HPE-2 (oePOSTN) group compared to the controls (Figure S11). These findings indicate that CAF-derived POSTN enhances EMT and tumor invasiveness *in vivo* by activating integrin β5-mediated PI3K/AKT/β-catenin signaling, consistent with the *in vitro* results.

### Pharmacological inhibitors of integrin αvβ5 partially reversed POSTN-induced proliferation, colony formation, migration and invasion of PDAC cells

**Figure 10:**
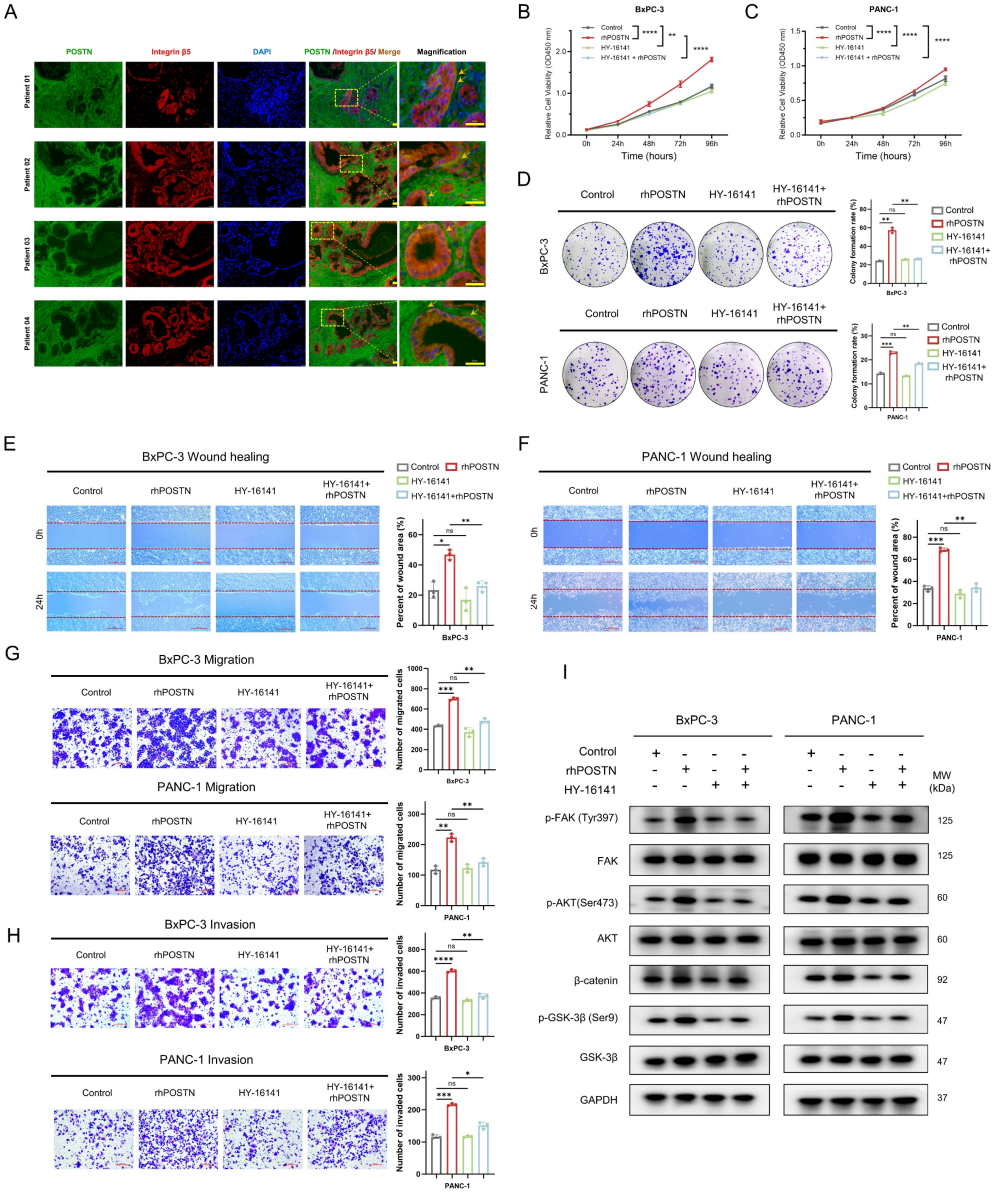
** Integrin αvβ5 inhibitors partially reverse POSTN-induced proliferation, colony formation, migration, and invasion of PDAC cells. (A)** Co-localization of POSTN and intergrin β5 in PDAC tissues. Immunofluorescence staining showing POSTN (green), integrin β5 (red), and nuclei (blue) in PDAC patient resection specimens. Scale bars, 25 μm. **(B-C)** Effect of integrin αvβ5 inhibition on POSTN-induced proliferation in BxPC-3 and PANC-1 cells. Cells were treated with: (1) negative control, (2) 500 ng/mL rhPOSTN, (3) integrin αvβ5 inhibitor (HY-16141) at 1/5 IC_50_ concentration, or (4) integrin αvβ5 inhibitor pretreated for 24 hours, followed by 500 ng/mL rhPOSTN. Cell proliferation was assessed using CCK-8 assays. **(D)** Colony formation assays in BxPC-3 and PANC-1 cells following the same treatments as in (B-C). **(E-F)** Wound healing assays in BxPC-3 and PANC-1 cells after treatments as described in (B-C), assessing migration capacity. **(G-H)** Transwell assays in BxPC-3 and PANC-1 cells to assess migation (G) and invasion (H) under the same treatment as in (B-C). **(I)** Western blot analysis of β-catenin expression, and phosphorylation of FAK, AKT, and GSK-3β in BxPC-3 and PANC-1 cells after 24-hour treatment as described in (B-C).

Based on the CellChat analysis-predicted POSTN-ITGAV/ITGB5 signaling axis, the expression correlation and functional significance of this axis in PDAC were further explored. Analysis of TCGA-PAAD RNA-seq data revealed strong positive correlations between *POSTN* and its receptors, *ITGAV* and *ITGB5*, at the mRNA level (Figure S12A-B). Spatial validation through immunofluorescence in tissue sections from four patients in the PUCH-PDAC cohort showed significant co-localization of POSTN and integrin β5 on tumor cell membranes, providing *in situ* evidence for the functional relevance of the POSTN-ITGAV/ITGB5 axis in PDAC (Figure 10A). At the cellular level, immunocytochemistry (ICC) confirmed that integrin αvβ5 localized predominantly on the surface of BxPC-3 and PANC-1 cells, supporting its role in mediating downstream signaling events (Figure S12C).

After building on the above expression correlation and spatial localization, the effect of blocking the POSTN-integrin αvβ5 axis on PDAC cell malignancy was assessed. To optimize the experiment and reduce the direct inhibitory effect of the integrin αvβ5 inhibitor (HY-16141), the working concentration for each cell line was determined according to its specific IC_50_ value (Figure S12D-E). The integrin αvβ5 inhibitor (HY-16141) markedly reversed rhPOSTN-induced proliferation, colony formation, migration, and invasion in BxPC-3 and PANC-1 cells (Figure 10B-H). To further examine the effects of the integrin αvβ5 inhibitor on POSTN-mediated activation of the PI3K/AKT/β-catenin signaling pathway in BxPC-3 and PANC-1 cells, Western blot analysis was conducted and revealed that rhPOSTN treatment alone significantly increased β-catenin levels and enhanced the phosphorylation of FAK, AKT, and GSK-3β when compared to the control group (Figure 10I). In contrast, treatment with the reduced concentration of the integrin αvβ5 inhibitor alone did not significantly affect these signaling molecules. Notably, co-treatment with rhPOSTN and the inhibitor (HY-16141) significantly reversed the rhPOSTN-induced increases in β-catenin levels and the phosphorylation of FAK, AKT, and GSK-3β (Figure 10I). These findings indicate that the integrin αvβ5 inhibitor can suppress POSTN-induced activation of the PI3K/AKT/β-catenin signaling pathway, further underscoring its role in mitigating POSTN-driven malignant phenotypes in PDAC cells.

## Discussion

Patients with PDAC continue to endure dismal survival rates, primarily owing to the intricate tumor microenvironment (TME)^31^. A significant feature of this environment is the desmoplastic stroma containing CAFs and ECM, which significantly contributes to tumor progression and treatment resistance 32. This emphasizes the critical need to explore CAFs in the context of PDAC. While several studies have investigated the heterogeneity of CAFs in PDAC at a single-cell resolution 33, 34, these efforts have been hindered by limitations in sample size and insufficient exploration of CAF-tumor cell interactions and clinically relevant targets. To address this, we conducted a comprehensive analysis of large-scale scRNA-seq, ST, and bulk RNA-seq data to delineate the heterogeneity of CAFs and epithelial cells in PDAC. Analysis of cell communication revealed a critical interaction between ECM-remodeling fibroblasts and EMT-related epithelial cells, specifically identifying the POSTN-ITGAV/ITGB5 axis as a key mediator of PDAC progression. In our PUCH-PDAC cohort, we validated a significant correlation between CAF-derived POSTN expression and tumor malignancy, as well as worse clinical prognosis. Functional and mechanistic studies revealed that CAF-derived POSTN orchestrates a pro-tumorigenic microenvironment by enhancing PDAC cell proliferation, migration, and invasion through the activation of the PI3K/AKT/β-catenin signaling pathway via integrin αvβ5.

Although the heterogeneity of CAFs has been explored from a pan-cancer perspective 4, 35, 36, their intricate heterogeneity across diverse levels still prompted us to focus on the heterogeneity of fibroblasts in PDAC context 37. We identified 21,629 fibroblasts from integrated single-cell transcriptomic samples, providing more detailed insights into the distinct subtypes. ECM-remodeling fibroblasts constitute the largest subgroup, predominantly enriched in tumor tissues. These fibroblasts showed a marked increase during tumor progression, localized in close spatial proximity to tumor cells, and accumulated prominently at terminal positions in pseudotime trajectories. Further analysis of transcriptional regulation revealed elevated expression and activity of SOX11 in these fibroblasts, which was significantly correlated with poor prognosis. This finding offers valuable insights for future research and potential therapies, although further experiments are needed to understand how SOX11 drives ECM-remodeling remodeling.

PDAC exhibit diverse cellular states that contribute to intratumoral heterogeneity (ITH), posing a significant hurdle for effective therapeutics 19. Traditional molecular classifications based on bulk RNA-sequencing have primarily addressed intertumoral heterogeneity, categorizing tumors into "classical" and "basal-like" subtypes 27. Although the resolution of intratumoral heterogeneity has advanced markedly with single-cell transcriptomic technologies, comprehensive explorations specific to PDAC remain relatively limited 7, 8, 14, 16. Thus, we systematically delineated the diverse epithelial cellular states present in our established dataset of integrated PDAC single-cell transcriptomes, providing valuable insights into the transcriptional ITH of PDAC tissues. The epithelial cell states identified in our study provide a substantial extension of the previously established Moffitt molecular classification, contributing a deeper understanding of PDAC heterogeneity. TFF1^+^ epithelial cells align with the "classical" subtype, while EMT-related epithelial cells correspond to the basal-like subtype. Trajectory analysis, inferCNV analysis, and prognostic evaluation of gene signatures of each subpopulation indicated that EMT-related epithelial cells represent a more aggressive phenotype. More importantly, analysis of cell communication and spatial proximity highlights the significant crosstalk between ECM-remodeling fibroblasts and EMT-related epithelial cells in malignant samples, accentuating their impact on tumor progression.

The interplay between fibroblasts and epithelial cells is critical for tumor progression, with ligand-receptor interactions serving as key mediators of this crosstalk 11. In this study, we identified the POSTN-ITGAV/ITGB5 axis as a tumor-promoting signaling axis that mediates crosstalk between ECM-remodeling fibroblasts and EMT-associated epithelial cells. POSTN is a secretory extracellular matrix protein predominantly expressed by fibroblasts within the tumor stroma of PDAC tissues. Clinical validation using the PUCH-PDAC cohort revealed a strong association between elevated POSTN expression and poor prognosis, as well as malignant phenotypes in patients with PDAC. Similarly, previous studies on breast cancer have shown that serum POSTN levels correlate with tumor stage, lymph node involvement, and distant metastasis, highlighting its diagnostic potential, particularly when combined with biomarkers such as CA153 and CEA 38. These findings implicate POSTN as a key modulator of the TME and stromal-epithelial crosstalk, establishing it as a promising prognostic biomarker for PDAC. Further exploration of serum POSTN as a non-invasive biomarker could improve early detection and metastasis prediction, expanding its clinical utility. Functionally, *in vitro* experiments demonstrated that POSTN promotes EMT in PDAC cells by activating the FAK/AKT/β-catenin signaling pathway, driving proliferation, migration, and invasion. Supporting evidence from glioblastoma stem cells shows that POSTN enhances self-renewal and malignancy via activation of the αvβ3/PI3K/AKT/β-catenin/FOSL1 pathway, upregulating key stemness markers such as CD133, Nestin, and SOX2^39^. Similarly, in hepatocellular carcinoma (HCC), POSTN facilitates the generation and maintenance of CD133+ liver cancer stem cells 40. These findings underscore the conserved role of POSTN in promoting cancer stemness and progression across malignancies. Crucially, POSTN sustains the stem-like properties of tumor cells, contributing to chemoresistance by enhancing their survival mechanisms and transcriptional plasticity, enabling the evasion of therapeutic interventions. From a therapeutic perspective, our study demonstrates that targeting integrin receptors that mediating POSTN signaling effectively disrupts POSTN-induced malignancies. Furthermore, pharmacological inhibition of the POSTN/TGFβ1/AP-2α axis with cilengitide, an αvβ3 integrin antagonist, combined with lenvatinib, an HCC first-line therapy, significantly enhanced therapeutic efficacy in preclinical models^40^. These findings link POSTN-integrin signaling to tumor progression and resistance, underscoring its clinical potential as a therapeutic target to suppress tumor growth, reduce cancer stemness, and improve treatment outcomes.

This study has several limitations. First, the scRNA-seq and ST data were sourced from public datasets. Although batch correction and validation datasets improved the credibility, additional in-house scRNA-seq and ST data are required for further confirmation. Second, although we performed detailed analyses of the major findings, some insights such as the role of SOX11 in ECM-remodeling fibroblasts were only profiled at a single-cell resolution without extensive functional validation. Nevertheless, these observations remain valuable as references for future research, upon which we aim to expand.

In conclusion, this study deepens understanding of PDAC by dissecting heterogeneity of cancer-associated fibroblast and epithelial cell using integrated single-cell and spatial transcriptomic approaches. Identification of the POSTN-ITGA5/ITGB5 signaling axis and its role in PDAC progression through the PI3K/AKT/β-catenin pathway elucidates key mechanisms underlying tumor aggressiveness and poor prognosis. Furthermore, our findings highlight the critical crosstalk between ECM-remodeling fibroblasts and EMT-associated epithelial cells, highlighting their potential as therapeutic targets. These insights not only advance the knowledge of PDAC pathophysiology but also provide a foundation for developing targeted therapies aimed at the TME, offering hope for improving clinical outcomes in this challenging malignancy.

## Supplementary Material

Supplementary figures.

Supplementary tables.

## Figures and Tables

**Table 1:** Correlation between stromal POSTN expression and clinicopathological characteristics in 173 patients from PUCH-PDAC cohort.

Characteristics	Patients, *n*=173 (%)	POSTN expression		*P* value
		High-group *n* =111 (%)	Low-group *n* =62 (%)	
Age at diagnosis (years)				0.357
<60	75 (43.4)	51 (68.0)	24 (32.0)	
>=60	98 (56.6)	60 (61.2)	38 (38.8)	
Gender				0.902
Male	91(52.6)	58 (63.7)	33 (36.3)	
Female	82 (47.4)	53 (64.6)	29 (35.4)	
Tumor differentiation				0.006
Well-differentiated	10 (5.9)	2 (20.0)	8 (80.0)	
Moderately-differentiated	147 (87.0)	95 (64.6)	52 (35.4)	
Poor-differentiated	15 (8.9)	13 (86.7)	2 (13.3)	
Unknown	1	1	0	
TNM stage				< 0.001
Ⅰ	28 (16.3)	10 (35.7)	18 (64.3)	
Ⅱ	103 (60.2)	64 (62.1)	39 (37.9)	
Ⅲ	13 (7.6)	12 (92.3)	1 (7.7)	
IV	28 (16.4)	25 (89.3)	3 (10.7)	
Unknown	1	0	1	
Tumor Size				0.006
≤ 2cm	15 (8.7)	4 (26.7)	11 (73.7)	
> 2cm, ≤ 4cm	89 (51.4)	61 (68.5)	28 (31.5)	
> 4cm	69 (39.9)	46 (66.7)	23 (33.3)	
Lymph node metastasis				< 0.001
Negative	74 (42.8)	32 (43.2)	42 (56.8)	
Positive	99 (57.2)	79 (79.8)	20 (20.2)	
Nervous invasion				0.421
Negative	18 (10.4)	10 (55.6)	8 (44.4)	
Positive	155 (89.6)	101 (65.2)	54 (34.8)	
Vascular invasion				0.003
Negative	97 (56.1)	53 (54.6)	44 (45.4)	
Positive	76 (43.9)	58 (76.3)	18 (23.7)	
Distant metastasis				0.002
Negative	145 (83.8)	86 (59.3)	59 (40.7)	
Positive	28 (16.2)	25 (89.3)	3 (10.7)	
Tumor site				0.698
Head	77 (44.5)	48 (62.3)	29 (37.7)	
Body and tail	95 (54.9)	62 (65.3)	33 (34.7)	
Whole pancreas	1 (0.6)	1	0	

**Table 2:** Cox proportional hazards regression model analysis of overall survival in PDAC patients.

Variables	Categories	Overall survival			
		Univariate analysis		Multivariate analysis	
		HR (95% CI)	*P* value	HR (95% CI)	*P* value
Age (years)	<60	Reference			
	>=60	1.20 (0.85-1.68)	0.299		
Gender	Male	Reference			
	Female	0.93(0.67-1.31)	0.689		
Tumor differentiation	Well-differentiated	Reference		Reference	
	Moderately-differentiated	5.52 (1.75-17.37)	0.004	4.74 (1.50-15.03)	0.008
	Poorly-differentiated	5.73 (1.61-20.36)	0.007	4.08 (1.10-15.09)	0.036
TNM stage	I	Reference		Reference	
	II	1.39 (0.86-2.24)	0.184	0.42 (0.41-3.24)	0.406
	III	1.37 (0.64-2.92)	0.414	0.50 (0.07-3.75)	0.498
	IV	2.92 (1.63-5.22)	<0.001	0.31 (0.04-2.67)	0.288
Tumor size	<=2cm	Reference		Reference	
	>2cm, <=4cm	1.68 (0.86-3.26)	0.126	1.37 (0.67-2.81)	0.385
	>4cm	2.06 (1.05-4.03)	0.036	1.44 (0.68-3.05)	0.339
Lymph node metastasis	Negative	Reference		Reference	
	Positive	1.46 (1.04-2.05)	0.028	0.92 (0.60-1.44)	0.92
Nervous invasion	Negative	Reference			
	Positive	1.16 (0.66-2.01)	0.610		
Vascular invasion	Negative	Reference		Reference	
	Positive	1.73 (1.23-2.44)	0.002	1.63 (1.09-2.44)	0.017
Distant metastasis	Negative	Reference		Reference	
	Positive	2.26 (1.47-3.47)	<0.001	9.71 (0.09-5.69)	0.744
Tumor site	Head	Reference			
	Body and tail	1.12 (0.80-1.57)	0.495		
	Whole pancreas	6.40 (0.86-47.60)	0.070		
POSTN expression	Lowly expressed	Reference		Reference	
	Highly expressed	2.09 (1.44-3.03)	<0.001	1.71 (1.09-2.68)	0.019

## Abbreviations

PDAC: Pancreatic ductal adenocarcinoma
CAF: Cancer-associated fibroblasts
EMT: epithelial-mesenchymal transition
ECM: extracellular matrix
scRNA-seq: single-cell RNA sequencing
GEO: Gene Expression Omnibus
HVG: highly variable gene
PCA: principal component analysis
UMAP: uniform manifold approximation and projection (UMAP)
ST: spatial transcriptomics
GO: Gene Ontology
GSVA: Gene set variation analysis
TF: Transcription factor
NMF: non-negative matrix factorization
MP: meta-program
CNV: Copy number variation
DEGs: Differential expressed genes
IHC: immunohistochemical (IHC)
ATCC: American type culture collection
CM): conditioned medium
GSEA: Gene Set Enrichment Analysis
KEGG: Kyoto Encyclopedia of Genes and Genomes
OS: Overall survival
DFS: Disease-free survival
Fibro: Fibroblast
Epi: Epithelium
rhPOSTN: recombinant human POSTN protein
TME: Tumor microenvironment
