# Supplementary material for: Integrative Single-Cell and Spatial Transcriptomics Analysis Reveals ECM-remodeling Cancer-associated Fibroblast-Derived POSTN as a Key Mediator in Pancreatic Ductal Adenocarcinoma Progression
Source: Int J Biol Sci. 2025 May 27;21(8):3573–96. doi: 10.7150/ijbs.108618 (PMC12160862; doi:10.7150/ijbs.108618)
Supplement: Supplementary file 1 — Supplementary figures. [file ijbsv21p3573s1.pdf]

Supplementary Content

Integrative Single-Cell and Spatial Transcriptomics Analysis Reveals ECM-remodeling Cancer-associated Fibroblast-Derived POSTN as a Key Mediator in Pancreatic Ductal Adenocarcinoma Progression

Supplementary Methods

1. Immunocytochemistry

BxPC-3 and PANC-1 cells were seeded in confocal 24-well plates (Nest, 801006) and cultured until reaching approximately 60% confluence. The cells were then fixed with cold acetone (−20°C) for 15 minutes. After blocking with 3% (w/v) BSA for 1 h at room temperature, the cells were incubated with primary antibodies anti-integrin αvβ5 (Sigma, MAB1961, 1:500) at 4 °C overnight. After be washed three times with PBS, the cells were incubated with the corresponding FITC-labeled secondary antibody (ZSGB-BIO, ZF-0312) at room temperature for 1 h. Finally, the nuclei were stained with DAPI (Sigma-Aldrich, St. Louis, MO, USA). Fluorescence images were captured using a laser scanning confocal microscope (Leica, TCS SP8 X).

2. Enzyme-linked immunosorbent assay (ELISA)

The concentration of POSTN protein (Periostin) in the CAF-tumor indirect co-culture system was detected using ELISA assays. When BxPC-3 cells cultured in a 10-cm dishes reached approximately 70% confluence, 40% CAF-oePOSTN conditioned medium (CM) or CAF-NC CM was added, and the cells were indirectly co-cultured for 24 h. Similarly, when PANC-1 cells in a 10-cm dishes reached approximately 70% confluence, 40% pCAF-shPOSTN CM or pCAF-NC CM was added for indirect co-culture for 24 h. Subsequently, 1 mL of the cell culture supernatant was collected, centrifuged, and subjected to ELISA analysis. POSTN levels were measured using a human POSTN ELISA kit (SEH339Hu, Cloud-Clone Corp.), according to the manufacturer’s instructions. Absorbance values were detected at 450 nm using a microplate reader.

Supplementary Tables

- Table S1 Clinical characteristics of samples integrated in the discovery cohort.
- Table S2 Data sources of samples in the validation cohort.
- Table S3 Data sources of spatial transcriptomics samples included in the study.
- Table S4 Clinical characteristics of the patients in PUCH-PDAC cohort (n =173).
- Table S5 Key reagents and corresponding source used in the study
- Table S6 The proportion of major cell types in all each individual samples in the discovery cohort.
- Table S7 The top 30 marker genes of fibroblast subclusters in the discovery cohort.
- Table S8 Transcription factor activities of each fibroblast subclusters.
- Table S9 Gene list for the epithelial meta-programs.
- Table S10 Top GO enrichment terms for meta-programs (MP1-MP14) extracted from the epithelial cells in the discovery cohort.
- Table S11 The top 30 marker genes of epithelial subclusters in discovery cohort.
- Table S12 Differentially expressed genes in fibroblasts based on SOX11 expression in the discovery cohort.
- TableS13 Differentially expressed genes in ductal epithelium based on CAF-Derived *POSTN* expression levels in the CRA001160 dataset.

Supplementary Figures

- Figure S1. Quality control and preprocessing of scRNA-seq data in the discovery cohort.
- Figure. S2. Quality control and preprocessing of scRNA-seq data in the validation cohort.

- Figure S3. Heterogeneity analysis of fibroblasts in the validation cohort
- Figure S4. Kaplan-Meier survival curves for overall survival of TCGA-PAAD patients stratified by the relative abundance of ECM-remodeling fibroblasts.
- Figure S5. GO enrichment analysis of significantly upregulated marker genes in each fibroblast subtype in the validation cohort.
- Figure S6. The evolutionary trajectories and transcriptional regulatory analysis of fibroblast subtypes in validation cohort.
- Figure S7. *SOX11* expression across all samples in the discovery cohort.
- Figure S8. Supplementary analytical information on the annotation of epithelial cell subpopulations.
- Figure S9. The selection process identifies POSTN as a potential target for subsequent validation and investigation.
- Figure S10. ELISA assay showing POSTN protein levels in the conditioned medium of the indirect coculture system of CCC-HPE-2 and BxPC-3 cells (A) and Primary CAF & PANC-1 cells (B)
- Figure S11. Representative images of IHC staining for Integrin β5, β-catenin (pathway marker), and EMT markers in xenograft tumor sections.
- Figure S12. Expression and localization of POSTN and integrin αvβ5 in PDAC cells and tissues, and determination of integrin αvβ5 inhibitor concentrations.

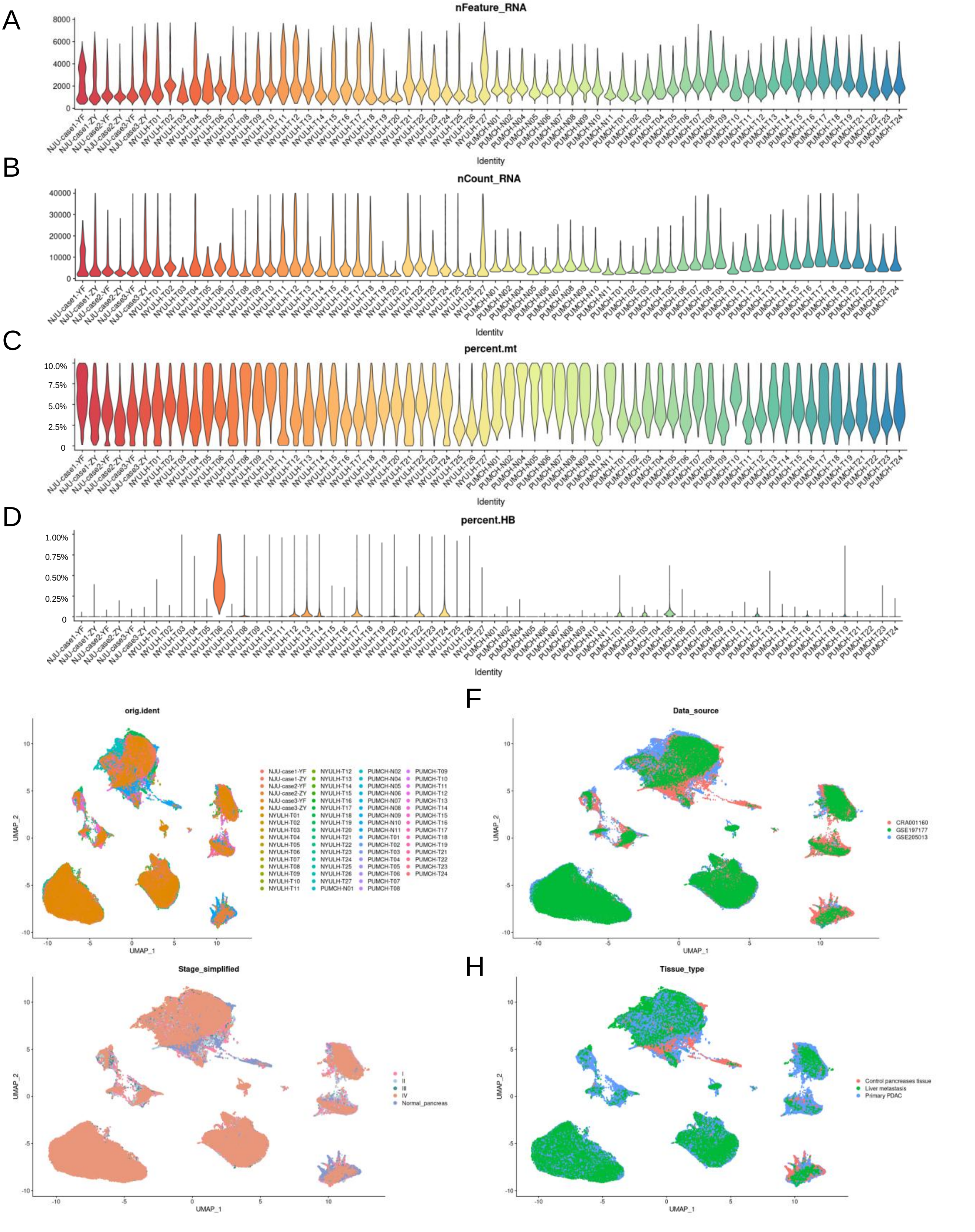

Figure S1. Quality control and preprocessing of scRNA-seq samples in the discovery cohort.

(A)-(D) Quality control metrics for scRNA-seq samples: nFeature_RNA (300-8,000), nCount_RNA (≤40,000), mitochondrial transcript percentage (≤10%), and hemoglobin transcript percentage (≤1%).

(E-H) The UMAP plot showing the distribution of cells from different samples, datasets, clinical stage, and tissue type after mitigating the impact of batch effect.

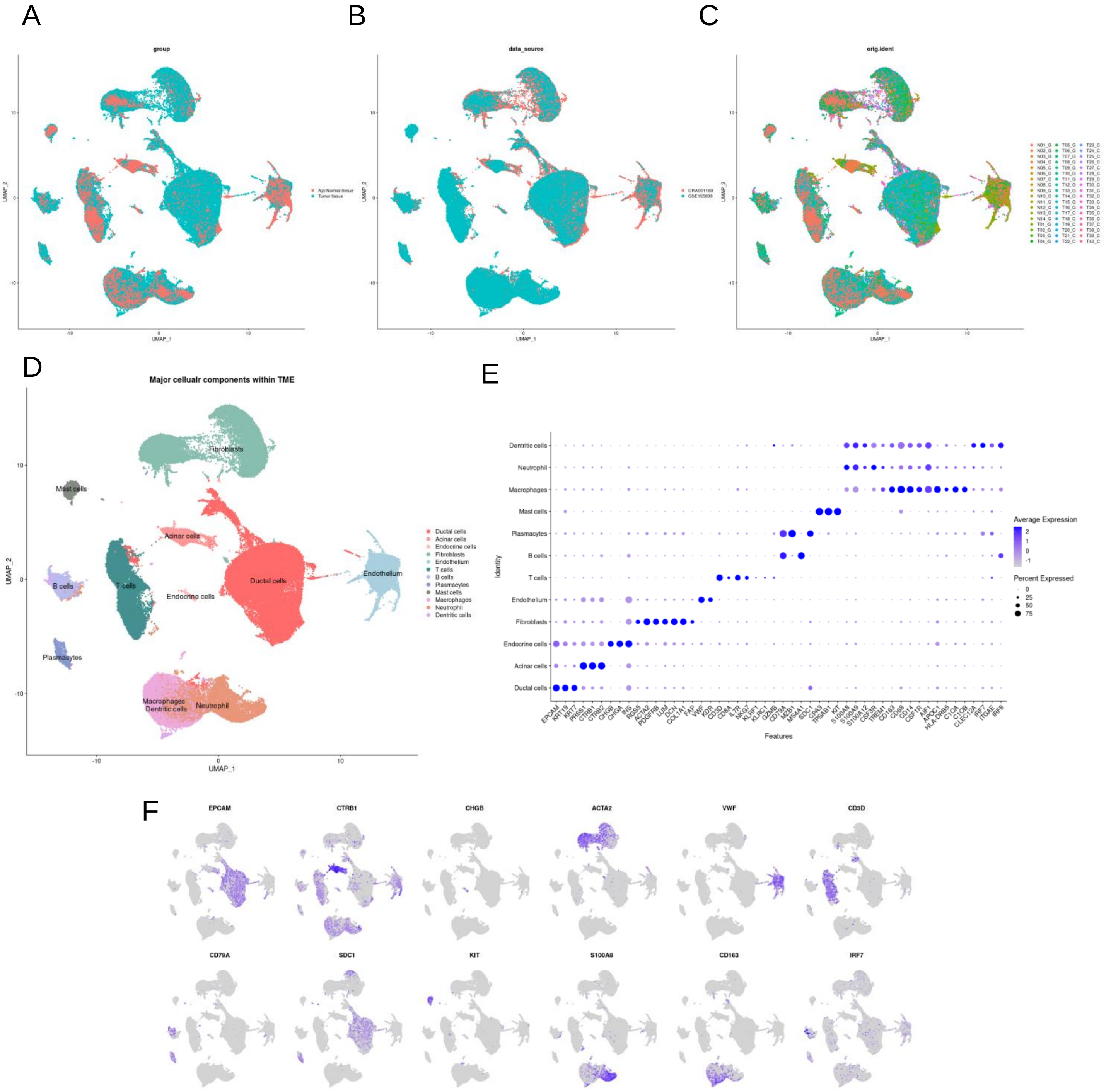

Figure. S2. Quality Control and Major Cell Type Clustering of scRNA-seq Data in the Validation Cohort.

(A-C) The UMAP plot showing the distribution of cells from different tissue types, data sources, and samples after mitigating the impact of batch effect.
(D) The UMAP plot showing the major cell types in validation cohort.
(E) Dot plot illustrating the marker genes utilized for the identification of major cell types.

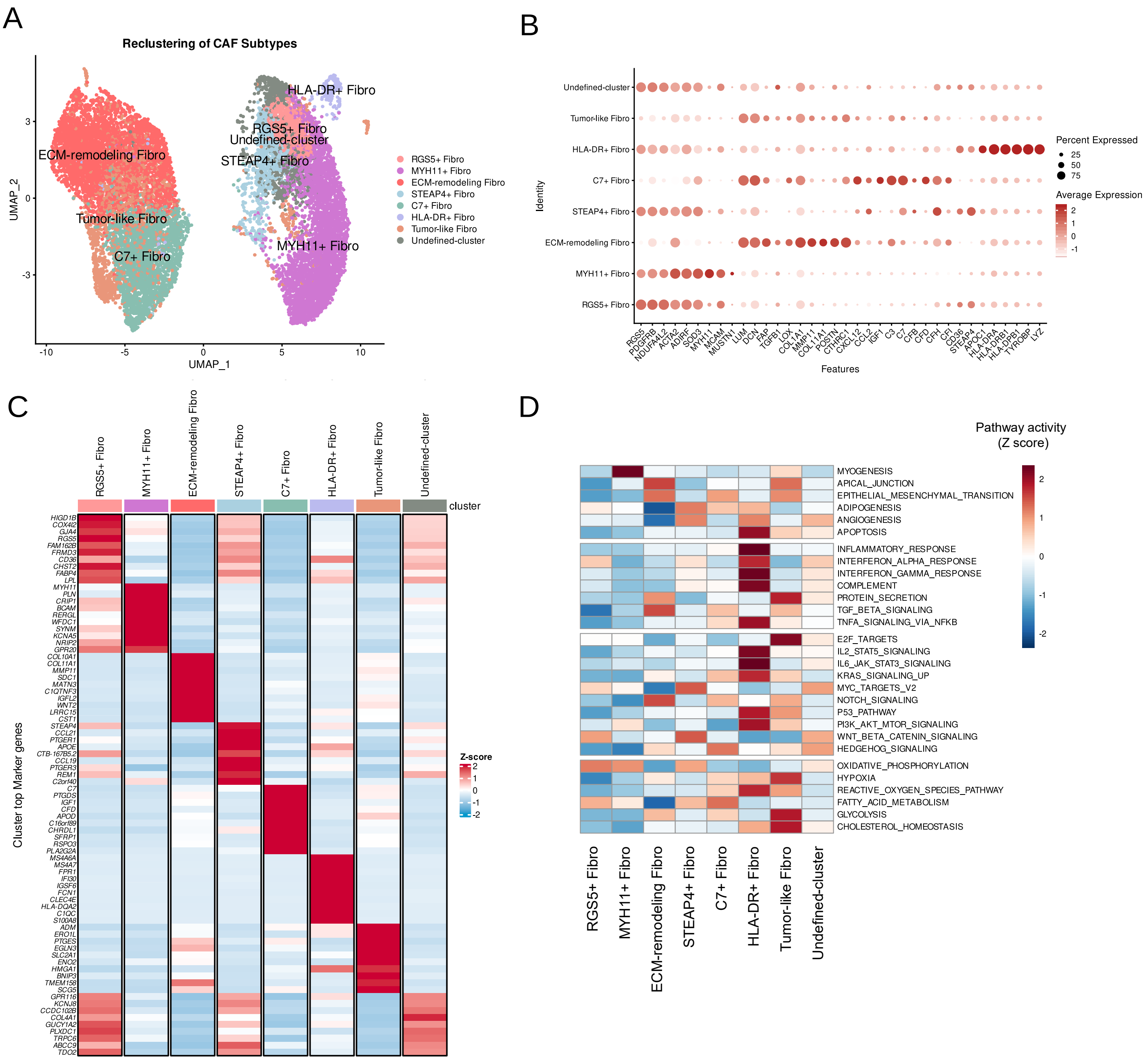

Figure S3. Heterogeneity analysis of fibroblasts in the validation cohort.

(A) UMAP plot showing the annotation of fibroblast subclusters in validation cohort, with different colors representing different subtypes.
(B) Dot plot showing the marker genes utilized for the identification of fibroblast subclusters.
(C) Heatmap showing the expression of the top ten differential expressed genes in the fibroblasts subclusters identified in validation cohort.
(D) Heatmap showing the pathway activities for each fibroblast subclusters scored by GSVA. Each column is normalized by z-score to indicate the relative pathway activities.

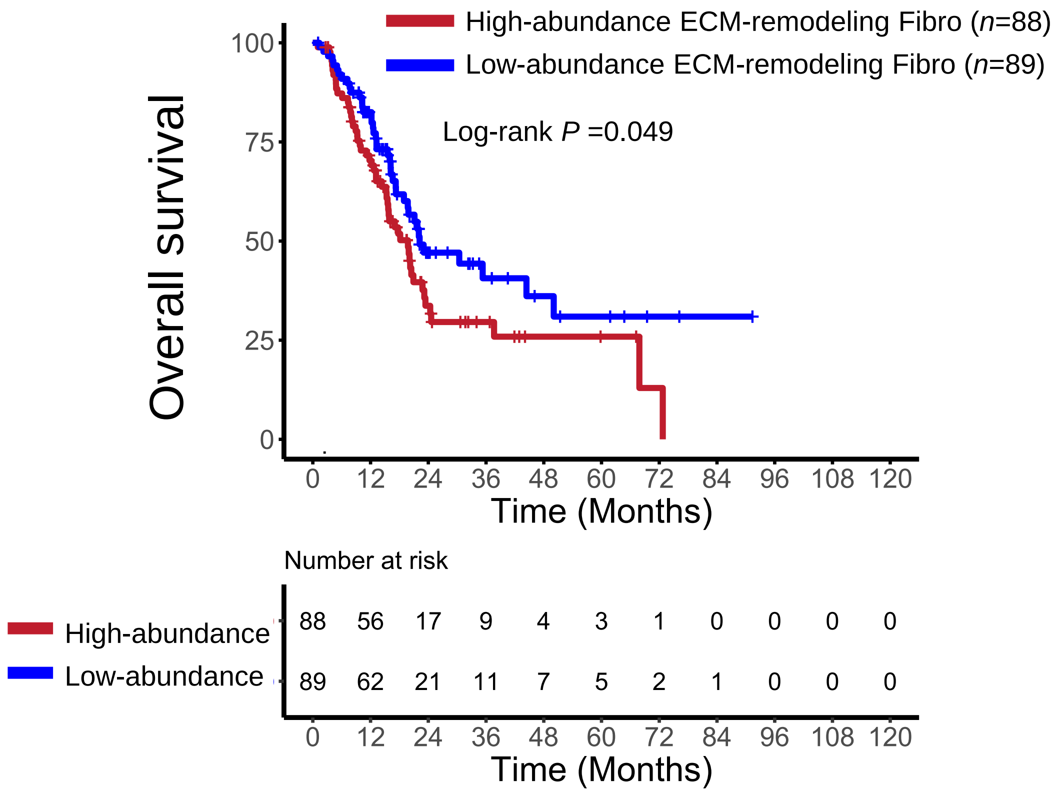

Figure S4. Kaplan-Meier survival curves for overall survival of TCGA-PAAD patients stratified by the relative abundance of ECM-remodeling fibroblasts.

Kaplan-Meier survival analysis for patients with PDAC was performed by dividing TCGA-PAAD bulk RNA-seq samples into high- and low-infiltration groups of ECM-remodeling fibroblasts based on their median abundance estimated using the CIBERSORTx algorithm.

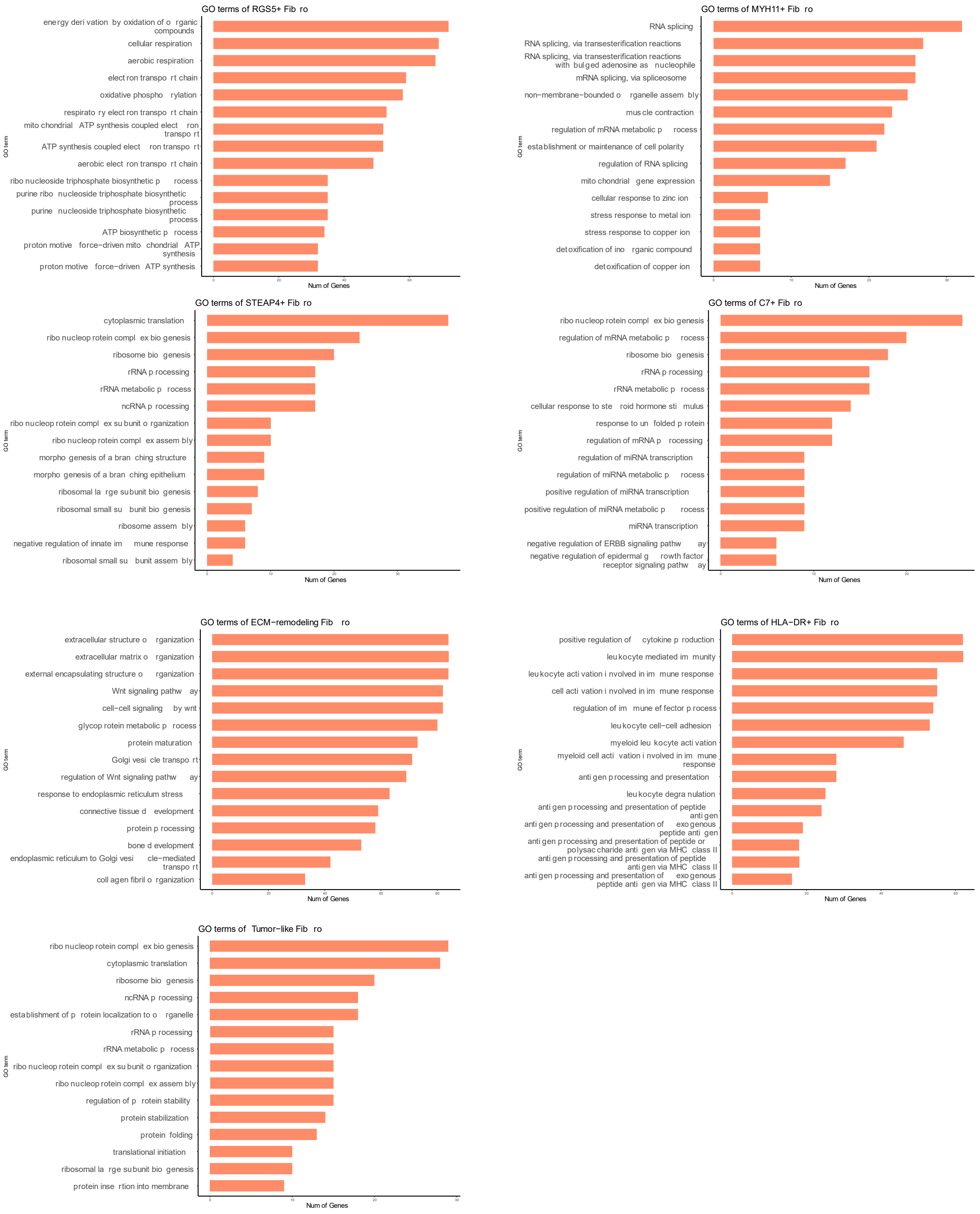

Figure S5. GO enrichment analysis of significantly upregulated marker genes in each fibroblast subtype in the validation cohort.

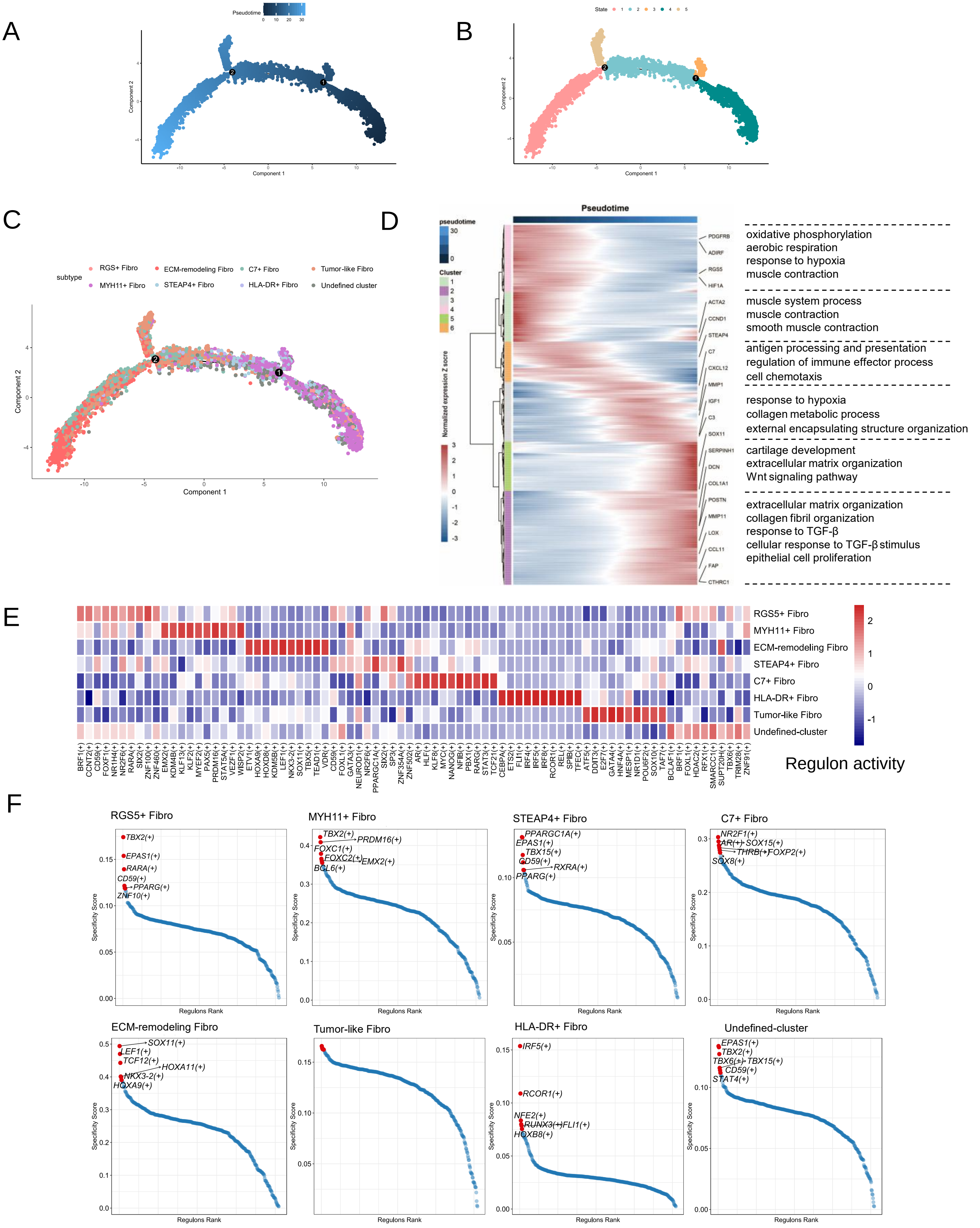

Figure S6. The evolutionary trajectories and transcriptional regulatory analysis of fibroblast subtypes in validation cohort. (A-C) Pseudotime analysis of fibroblast colored by pseudotime (A), state (B), and subtypes (C). (D) Heatmap showing scaled expression of DEGs along the pseudotime trajectory. (E) Heatmap showing the mean activity of top activated regulons in each fibroblast subtype. (F) Dot plot ranking the top differentially activated regulons in each fibroblast subtypes based on regulon-specific scores.

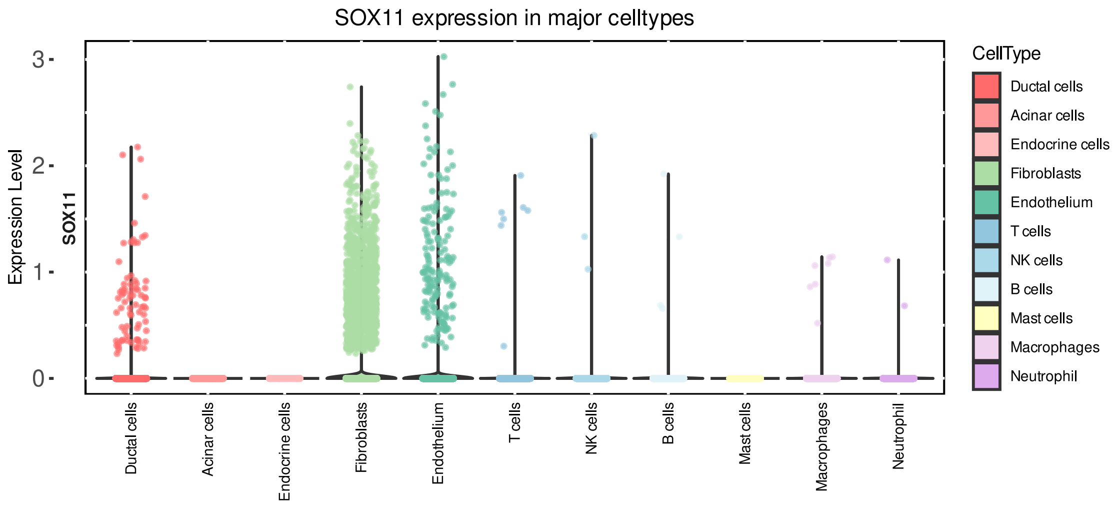

Figure S7. *SOX11* expression across all samples in the discovery cohort, grouped by major cell types.

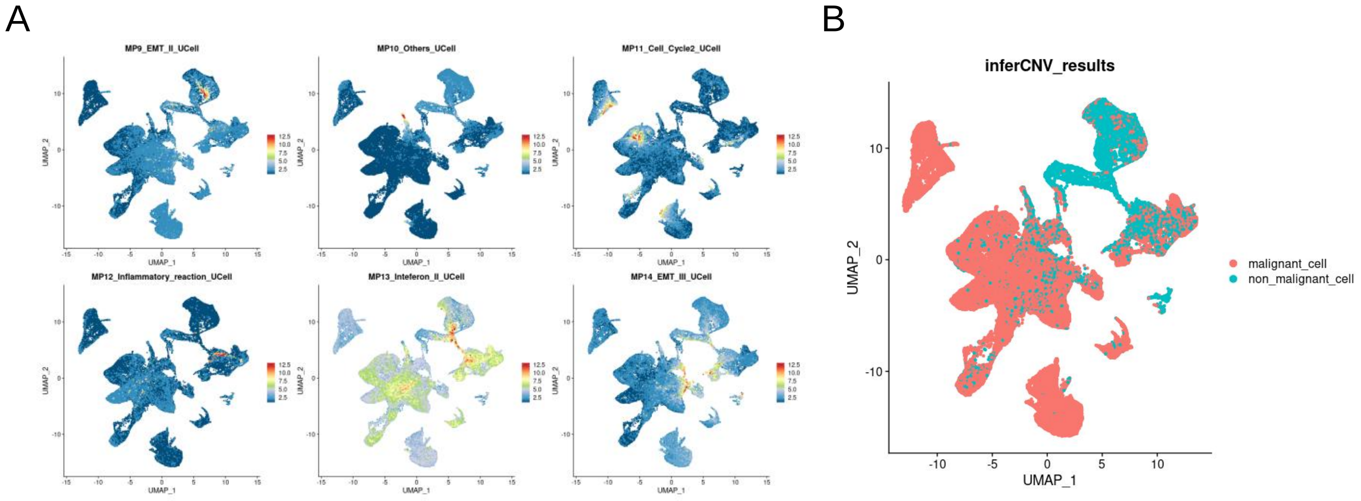

Figure S8. Supplementary analytical information on the annotation of epithelial cell subpopulations. (A) Feature plot of epithelial meta-program gene list scores calculated using the AddModuleScore function in Seurat (MP9-MP14). Colors indicate score levels, ranging from no expression (blue) to high expression (red). (B) The malignant and non-malignant status of epithelial cells inferred by the inferCNV analysis. All the above results are used to assist in the annotation of epithelial cell subpopulations.

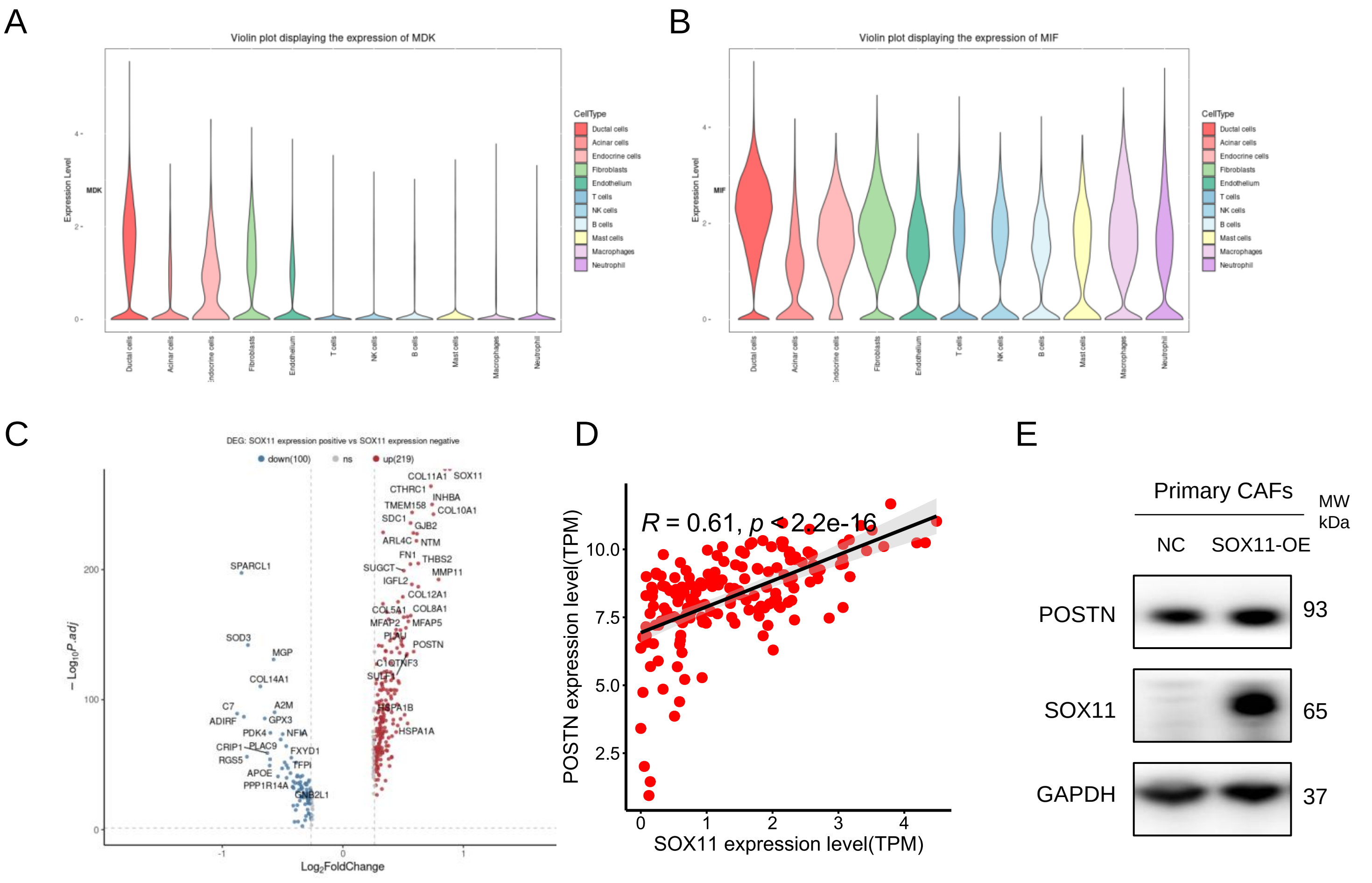

Figure S9. The selection process identifies POSTN as a potential target for subsequent validation and investigation. (A) Violin plot illustrating the expression of the candidate target *MDK* across all samples in the discovery cohort, stratified by major cell types. (B) Violin plot illustrating the expression of the candidate target *MIF* across all samples in the discovery cohort, stratified by major cell types. (C) Volcano plot displaying differentially expressed genes in fibroblasts grouped by SOX11 expression, highlighting *POSTN* as one of the most upregulated genes in the SOX11-high fibroblasts. (D) Correlation analysis of SOX11 and POSTN mRNA expression in the TCGA-PAAD dataset. (E) The overexpression efficiency of SOX11 in primary CAFs was evaluated by Western blot, and SOX11 overexpression significantly upregulated POSTN expression.

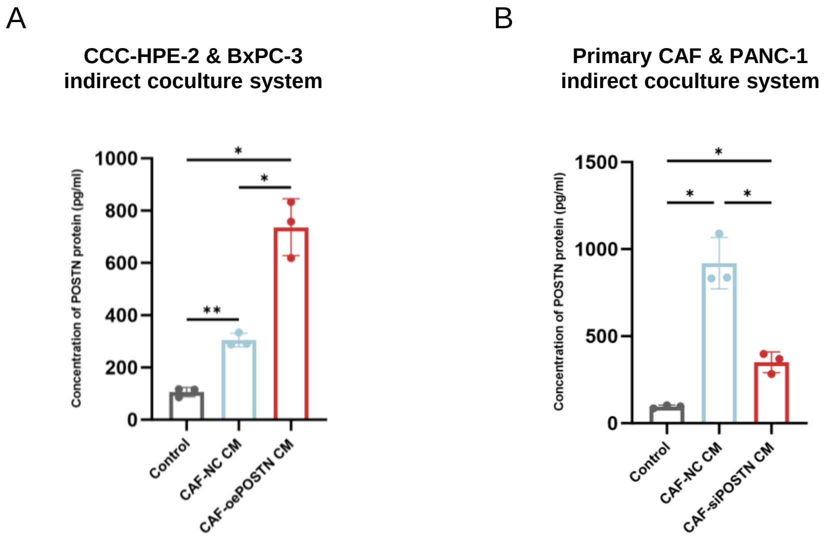

Figure S10. ELISA assay showing POSTN protein levels in the conditioned medium of the indirect coculture system of CCC-HPE-2 and BxPC-3 cells (A) and Primary CAF & PANC-1 cells (B)

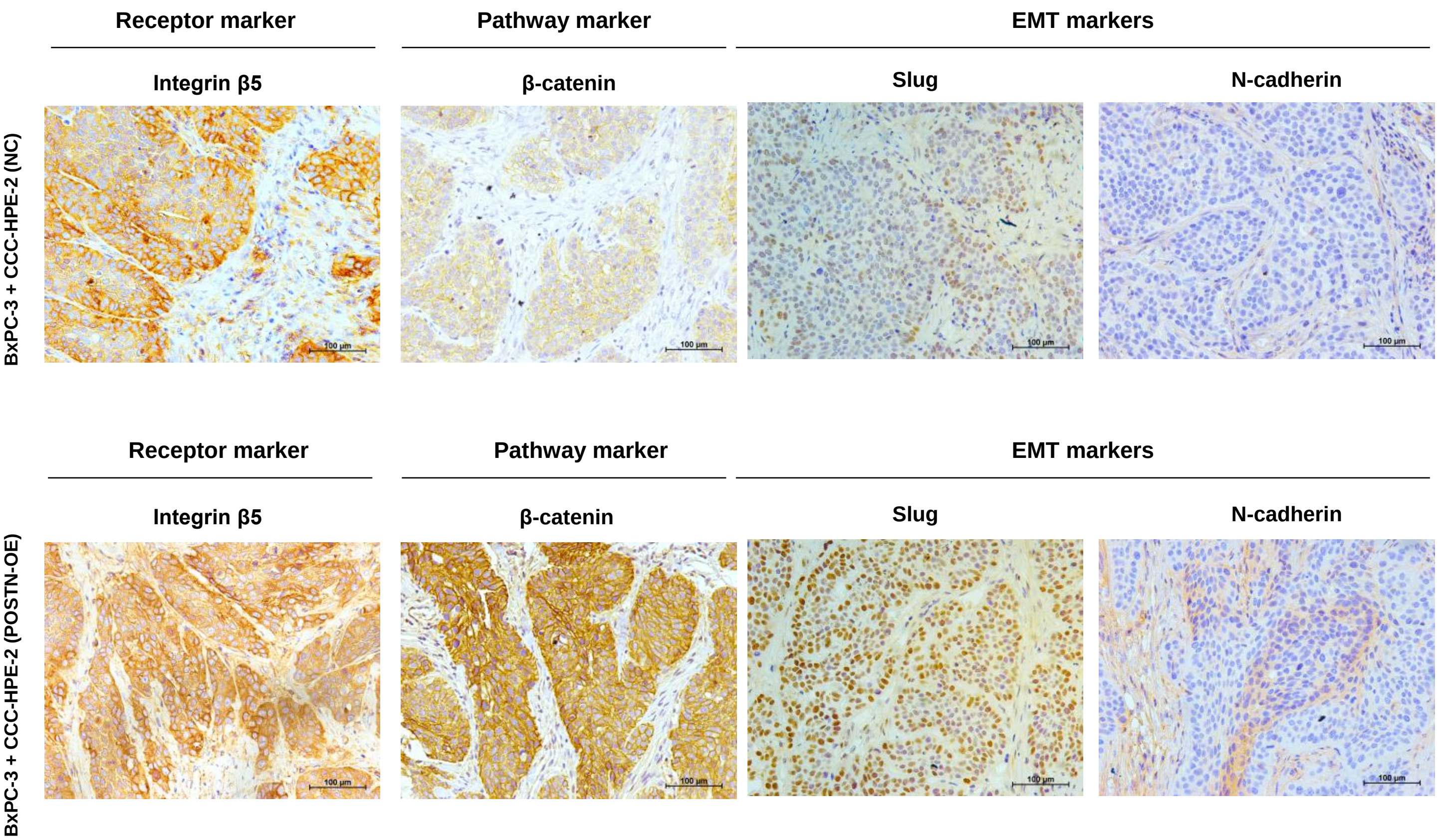

Figure S11. Representative images of IHC staining for Integrin β5, β-catenin (pathway marker), and EMT markers in xenograft tumor sections.

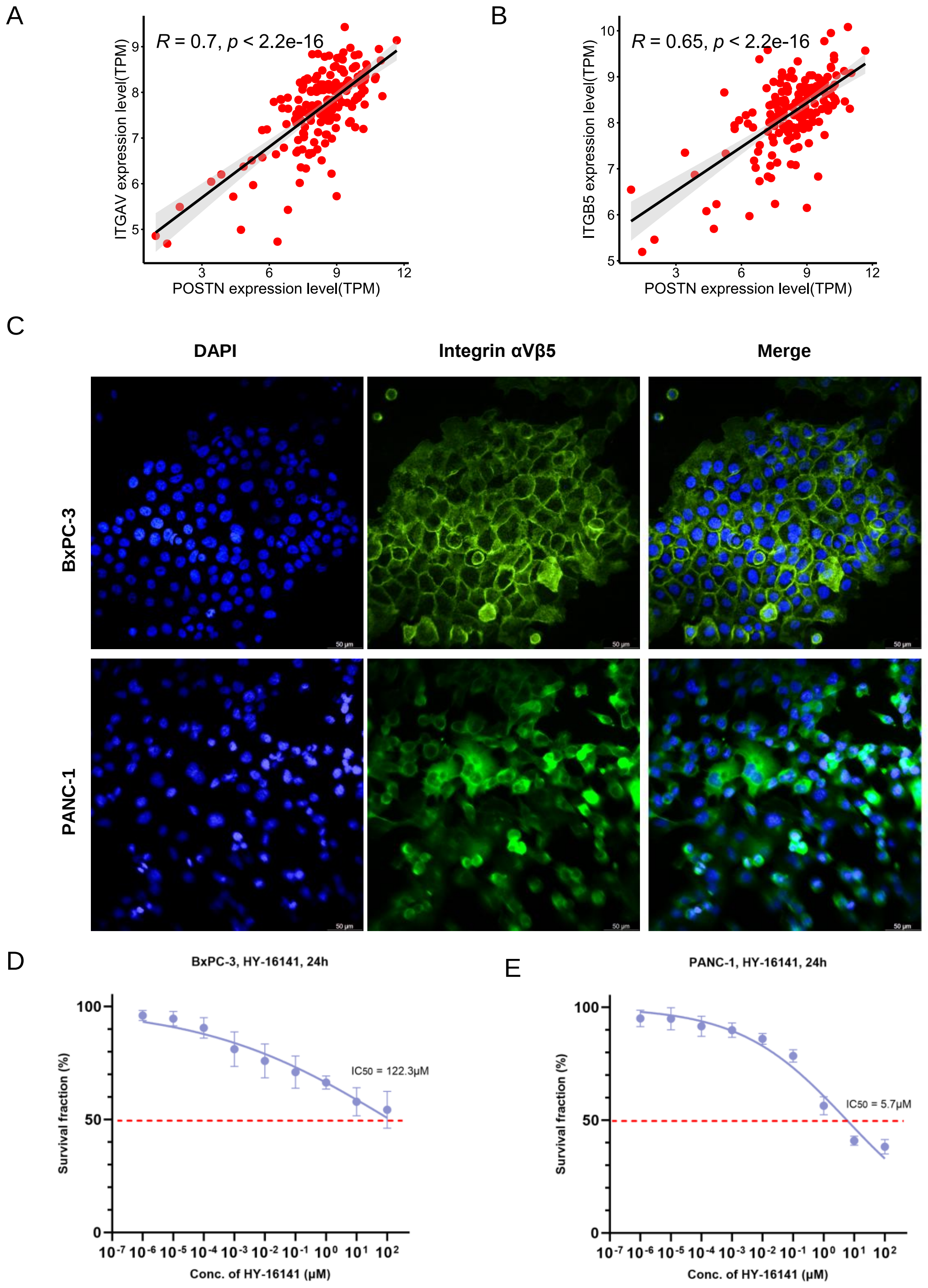

Figure S12. Expression and localization of POSTN and integrin αvβ5 in PDAC cells and tissues, and determination of integrin αvβ5 inhibitor concentrations

(A-B) Correlation analyses of *POSTN* and its receptors *ITGAV* and *ITGB5* at the mRNA level in PDAC, based on TCGA-PAAD RNA-seq dataset (n = 178).
(C) Immunofluorescence staining showing the surface localization of integrin αvβ5 on BxPC-3 and PANC-1 cells. Integrin αvβ5 is predominantly localized on the cell membrane in both cell lines.
(D-E) IC50 determination of the integrin αvβ5 inhibitor (HY-16141) in BxPC-3 and PANC-1 cells. Working concentrations for subsequent experiments were optimized based on IC50 values to reduce the direct inhibitory effects of the inhibitor.
